# Cardiac glycosides target barrier inflammation of the vasculature, meninges and choroid plexus

**DOI:** 10.1038/s42003-021-01787-x

**Published:** 2021-02-26

**Authors:** Deidre Jansson, Victor Birger Dieriks, Justin Rustenhoven, Leon C. D. Smyth, Emma Scotter, Miranda Aalderink, Sheryl Feng, Rebecca Johnson, Patrick Schweder, Edward Mee, Peter Heppner, Clinton Turner, Maurice Curtis, Richard Faull, Mike Dragunow

**Affiliations:** 1 Department of Pharmacology and Clinical Pharmacology, The University of Auckland, Auckland, New Zealand; 2 Centre for Brain Research, The University of Auckland, Auckland, New Zealand; 3 Department of Psychiatry and Behavioural Science, University of Washington, Seattle, WA USA; 4 Department of Anatomy and Medical Imaging, The University of Auckland, Auckland, New Zealand; 5 Center for Brain Immunology and Glia (BIG), Washington University, St. Louis, MO USA; 6 Department of Pathology and Immunology, School of Medicine, Washington University in St Louis, St. Louis, MO USA; 7 Department of Pathology, University of Otago, Christchurch, New Zealand; 8 Centre for Free Radical Research, University of Otago, Christchurch, New Zealand; 9 Department of Neurosurgery, Auckland City Hospital, Auckland, New Zealand; 10 Starship Hospital, Auckland, New Zealand; 11 Department of Anatomical Pathology, LabPlus, Auckland City Hospital, Auckland, New Zealand

**Keywords:** Drug development, Phenotypic screening

## Abstract

Neuroinflammation is a key component of virtually all neurodegenerative diseases, preceding neuronal loss and associating directly with cognitive impairment. Neuroinflammatory signals can originate and be amplified at barrier tissues such as brain vasculature, surrounding meninges and the choroid plexus. We designed a high content screening system to target inflammation in human brain-derived cells of the blood–brain barrier (pericytes and endothelial cells) to identify inflammatory modifiers. Screening an FDA-approved drug library we identify digoxin and lanatoside C, members of the cardiac glycoside family, as inflammatory-modulating drugs that work in blood–brain barrier cells. An ex vivo assay of leptomeningeal and choroid plexus explants confirm that these drugs maintain their function in 3D cultures of brain border tissues. These results suggest that cardiac glycosides may be useful in targeting inflammation at border regions of the brain and offer new options for drug discovery approaches for neuroinflammatory driven degeneration.

## Abstract (web summary)

Jansson et al. design a high content screening system to target inflammation in human brain cells of the blood–brain barrier (pericytes and endothelial cells) to identify inflammatory modifiers. They identify cardiac glycosides as powerful regulators of neuroinflammatory pathways in brain barrier tissues such as vasculature, meninges and choroid plexus.

## Introduction

Neuroinflammation contributes to many brain disorders with dysregulated chronic inflammatory processes occurring early in their progression, contributing to blood–brain barrier dysfunction and precipitating neurodegeneration^1–5^. Accumulating evidence suggests an inflammatory contribution by cells of the cerebrovasculature, meningeal compartments and choroid plexus (ChP), which facilitate immune cell trafficking across the barriers^6–8^. Since neuroinflammation in the central nervous system (CNS) can precede and exacerbate neuronal loss, attenuation of inflammation at these barrier tissues may reduce neurodegeneration^9–11^.

Neuroinflammation can be most easily targeted anatomically at several sites; the cerebrovasculature bordered luminally by the blood, and abluminally by the cerebrospinal fluid (CSF), the meninges which surround and envelop the brain and spinal cord directly communicating with lymphatic vessels and the ChP that produces CSF and facilitates fluid movement by way of glymphatic flux^12–14^. The brain’s immune functions have been concentrated at these border tissues, which may be more sensitive and more functionally primed to respond to inflammatory cues than the rest of the CNS^15–17^. We have designed our study to test inflammatory modulating drugs on the cell types that make up these structures.

At the cellular level the neurovascular unit (NVU) is formed by the specialised arrangement of endothelia, pericytes, astrocyte end-feet and neurons that work together to maintain brain homoeostasis. We have demonstrated that pericytes derived from post-mortem human brains display a robust inflammatory response when stimulated by classical inflammatory mediators such as tumour necrosis factor α (TNFα), interferon γ (IFNγ), interleukin 1β (IL-1β) and lipopolysaccharide (LPS), and are likely to be targets of neuroinflammation in both an autocrine and paracrine fashion^18,19^. Importantly, endothelia and pericytes in the NVU contribute to neuroinflammatory responses by chemokine secretion, adhesion molecule-mediated leucocyte extravasation and damage- and pathogen- associated molecular patterns receptor expression^12,20–23^. Attenuating the infiltration of peripheral immune cells into the brain or preventing inflammatory propagation by barrier cells such as neurovascular cells is likely to be beneficial in limiting CNS immune responses, although few CNS therapies have been based upon this approach. The drug natalizumab used in the treatment of multiple sclerosis that targets immune cell entry into the CNS is a good example of this approach^24^. In this study, in addition to primary human brain neurovascular cells such as pericytes, endothelia cells and microglia, we also made use of explants from barrier tissues such as meninges and ChP, which are directly involved in inflammation.

The human meninges are composed of three membranes (dura, arachnoid and pia mater) that envelop the brain and spinal cord. The arachnoid and pia mater, collectively termed the leptomeninges, form a semipermeable membrane to the CSF, which fills the subarachnoid space. The leptomeninges are comprised of several cell types, including macrophages, dendritic cells, mast cells and fibroblasts and are permeated by leptomeningeal arteries^25,26^. Furthermore, diverse leucocyte populations reside in the subarachnoid space providing a conduit for immune-brain communication^27–29^. Meningeal inflammation often precedes inflammation in the CNS and is present in neurodegenerative diseases, chronic inflammatory conditions and acute pathogen introduction^30–32^. Importantly, meningeal-derived factors can permeate the brain parenchyma, presumably through glymphatic exchange, suggesting that modulating meningeal-derived inflammation also represents an appropriate target to prevent inflammatory-mediated CNS insults^33,34^.

The ChP is a highly vascularised tissue that harbours fenestrated capillaries surrounded by apical facing, CSF-producing epithelial cells and serves as a gateway for immune cells from the circulatory system to the CNS^35^. The fact that the ChP is a sink for immune cells is of particular importance when it comes to immune cell trafficking in both sterile inflammatory and chronic inflammatory conditions^8,36^. Similarly, the ChP itself increases secretion of inflammatory messenger molecules into the CSF with age and disease, which are transported throughout the brain through circulation of CSF^37,38^. Together this supports the examination of the ChP as a target for reducing neuroinflammation.

Here, we utilise primary dissociated human brain pericytes and endothelia, as well as explants from the meninges and the ChP to screen drugs for anti-inflammatory properties. Drug screening approaches are not typically performed using primary human brain cells due to limited tissue yields, difficulties of human brain cell culture and accessibility. However, rodent immune cells display numerous discrepancies compared to their human counterparts, with respect to both immune functions and pharmacological responses^39,40^, suggesting that the use of primary human brain cells may identify compounds with greater translational impact. Primary human brain pericytes are suitable for high throughput drug screening for candidate compounds with anti-inflammatory functions because unlike many other human brain cell types, pericytes undergo rapid proliferation in vitro, allowing for efficient bulking of these cells for the purposes of high content drug screening. In this study, we screened human brain pericytes with a library of >1280 compounds for the ability to modify IL-1β responses. Selected hits from initial screens were validated in both pericyte and endothelial cultures to explore the anti-inflammatory efficacy of these compounds in NVU-associated cells. Next, we describe and characterise the inflammatory contribution of an ex vivo model of human leptomeningeal and ChP explants using it to investigate lead anti-inflammatory compounds in a complex multicellular system more closely recapitulating the in vivo human brain environment. Finally, we demonstrate the efficacy of two inflammatory modulating compounds digoxin and lanatoside C, in attenuating meningeal and ChP inflammatory responses and preventing subsequent inflammatory propagation.

## Results

### High content screen to identify inflammatory modulators in pericytes

**Fig. 1:**
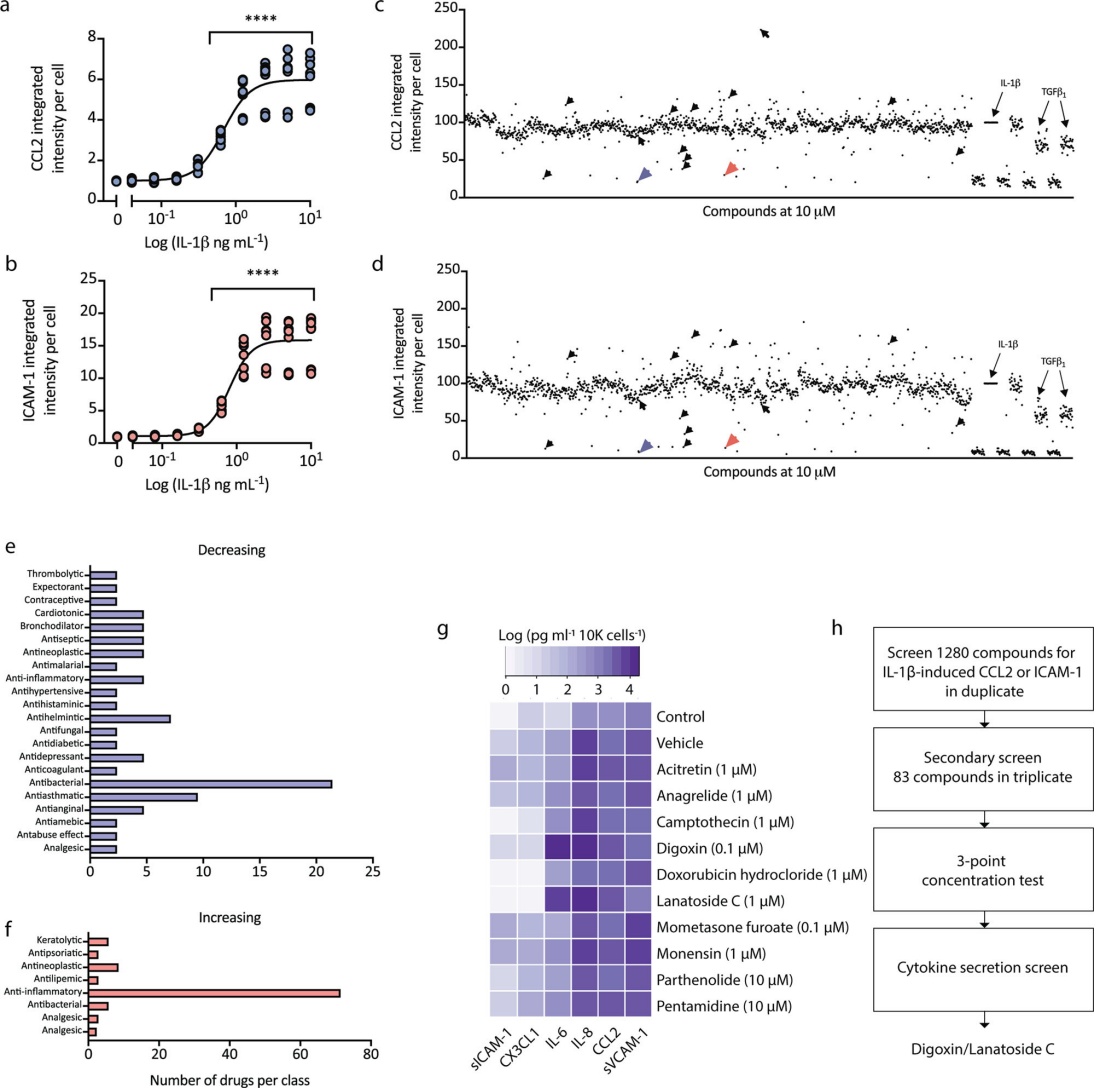
High throughput screening of pericytes identifies inflammatory-modulating compounds. **a, b** Pericytes were treated with IL-1β for 24 h and immunostained for CCL2 and ICAM-1 protein expression. Integrated intensity per cell was calculated using total cells (Hoechst), *n* = 3, significance determined using one-way ANOVA with Dunnett’s correction for multiple comparisons, mean ±  SEM.****p < 0.0001. Case used H239. **c**, **d** Pericytes were screened for compounds that can modify IL-1β-induced CCL2 or ICAM-1 expression using the Prestwick chemical library. Mean integrated intensity per cell was calculated using total cell counts and normalised to IL-1β + vehicle condition (arrow) (blue arrow-digoxin, red arrow-lanatoside C, black arrows-hit compounds tested in (**g**)). Internal controls were present on each drug plate (TGFβ_1_, 1 or 10 ng mL^−1^). Case used: H239. **e**, **f** Hits identified using cut-off criteria from primary screen that modified CCL2 or ICAM-1 expression with known therapeutic uses. **g** Conditioned media from pericytes pre-treated with hit compounds for 24 h, then stimulated with IL-1β (0.05 ng mL^−1^) for 24 h was analysed by CBA, data was normalised to cell number and presented as the logged value of cytokine secretion in pg/mL/10,000 cells. Control (0.01% BSA in PBS) + dimethylsulfoxide (DMSO), Vehicle (IL-1β (0.05 ng mL^−1^ + (DMSO)), remainder are IL-1β + drug (*n* = 2, media pooled from triplicate wells for analysis). Cases used E206, E213. **h** Drug screening pipeline in primary human brain pericytes. Initial screen in pericytes completed in duplicate wells and secondary screen in 83 compounds that met cut-off criteria was completed in triplicate wells. Effects that were reproduced were examined using a three-point concentration test, then tested for effects on cell secretion (data in Fig. S1).

Primary human brain pericytes respond to inflammatory stimuli to induce the expression of chemokines and adhesion molecules, including chemokine (C-C motif) ligand 2 (CCL2) and intercellular adhesion molecule-1 (ICAM-1), respectively^18,41–43^. Due to their involvement in enhancing leucocyte infiltration into the brain, these mediators were selected as candidate proteins to determine the anti-inflammatory potential of the Prestwick FDA-approved compound library^18^. To calibrate the assay, concentration-response curves of IL-1β-induced CCL2 and ICAM-1 protein expression, as determined by immunocytochemistry were undertaken and revealed an EC_50_ of 0.02 ng mL^−1^ for CCL2 and 0.03 ng mL^−1^ for ICAM-1, thus 0.05 ng mL^−1^ was selected as an optimal concentration of IL-1β to determine immune modulation, allowing for identification of compounds which may induce and attenuate inflammatory responses (Fig. 1a, b).

Pericytes were screened for modulators of IL-1β-induced CCL2 and ICAM-1 expression by pre-treating cells with >1200 FDA approved compounds each at 10 µM in duplicate for 24 h, then stimulating with IL-1β (0.05 ng mL^−1^) for 24 h. CCL2 and ICAM-1 intensity values normalised to total cell counts confirmed inhibitory effects of transforming growth factor receptor β (TGFβ_1_)_,_ a known inhibitor of chemokine and adhesion molecule expression in pericytes^43^ (Fig. 1c, d). Plates were internally normalised to account for plate variability in terms of cell numbers and absolute intensities, and the IL-1β induction of CCL2 or ICAM-1, and the TGFβ_1_-dependent inhibition was consistent across all test plates (Fig. S1). *Z*-scores^44^ were also calculated to measure plate variability for the CCL2 and ICAM1 induction, returning values of 0.43 and 0.15, respectively (Fig. S1). This analysis shows that CCL2 expression is more consistent across plates than ICAM1, and therefore a superior assay measure. Despite this difference in *Z*-scores when we correlated (using Pearson’s correlation coefficient) the normalised intensity values for CCL2 against ICAM1 for all drug treatments across all plates we observed a very strong positive correlation of *r* = 0.9508, *p* < 0.0001, suggesting that both measures were strongly associated with each other. A total of 82 compounds that met cut-off criteria were selected for further validation (see Methods). Drug classification revealed hit compounds that altered CCL2 or ICAM-1 were mainly comprised of therapeutics categorised as anti-bacterial, anti-asthmatic, anti-helminthic, cardiotonic, bronchodilator, anti-septic, anti-neoplastic, anti-amoebic and anti-inflammatory (Fig. 1e, f). A second confirmatory screen of the 82 hits in triplicate generated 44 that met cut-off criteria (see Methods). These 44 hits were then screened at three concentrations (0.1, 1 and 10 µM) for the ability to modify IL-1β-induced CCL2 or ICAM-1 in pericytes, without causing significant cell loss (Trial 3, Fig. S1). IL-1β-independent effects were assessed by screening compounds in pericytes with the same paradigm (24 h of compounds at 10 µM, 24 h of vehicle only for IL-1β), which revealed no significant changes relating to CCL2 or ICAM-1 expression (Fig. S1).

Hits were narrowed down to a more manageable ten compounds that consistently modified either IL-1β-induced CCL2 or ICAM-1 expression by immunocytochemistry without significant cell reduction (as assessed by total cell counts, Fig. 1g). These ten were selected on the basis of their efficacy as well as diversity in drug type. The anti-inflammatory effects of these ten were then studied more intensively, including their effects on secretions of a larger panel of inflammatory chemokines, cytokines and adhesion molecules previously identified to be secreted by pericytes in response to IL-1β as determined by cytometric bead array (CBA)^19^. Drug concentrations from Trial 3 that resulted in maximal changes in either CCL2 or ICAM-1 with the least cell loss were used to treat pericytes as in Fig. 1 (Fig. S1). Analysis of cytokine secretion showed that two cardiac glycosides digoxin and lanatoside C had the most substantial effects on soluble ICAM-1 (sICAM-1) and fractalkine (CX3CL1) (a known microglial ligand) expression^45^. In particular, digoxin inhibited IL-1β-induced secretion of CCL2, sICAM-1, soluble vascular cell adhesion molecule-1 (sVCAM-1), and CX3CL1 but increased secretion of interleukin-6 (IL-6) more than 6-fold over IL-1β alone (Fig. 1g, Fig. S2). A generalised pipeline for refinement of the hit compound list leading to digoxin as a lead compound is presented in Fig. 1h. Digoxin is indicated for the treatment of symptomatic heart failure, and atrial fibrillation, thus we were surprised to see anti-inflammatory effects on vascular cells. Lanatoside C, another cardiac glycoside found in the Prestwick library, demonstrates structural similarity with digoxin^46^. Little is known concerning inflammatory outputs by cardiac glycosides on brain vascular cells, and due to the ability of digoxin to most effectively limit pericyte-mediated inflammatory responses, digoxin and the closely-related lanatoside C were chosen as lead compounds for further analysis.

### Effects of digoxin and lanatoside C in pericytes

**Fig. 2:**
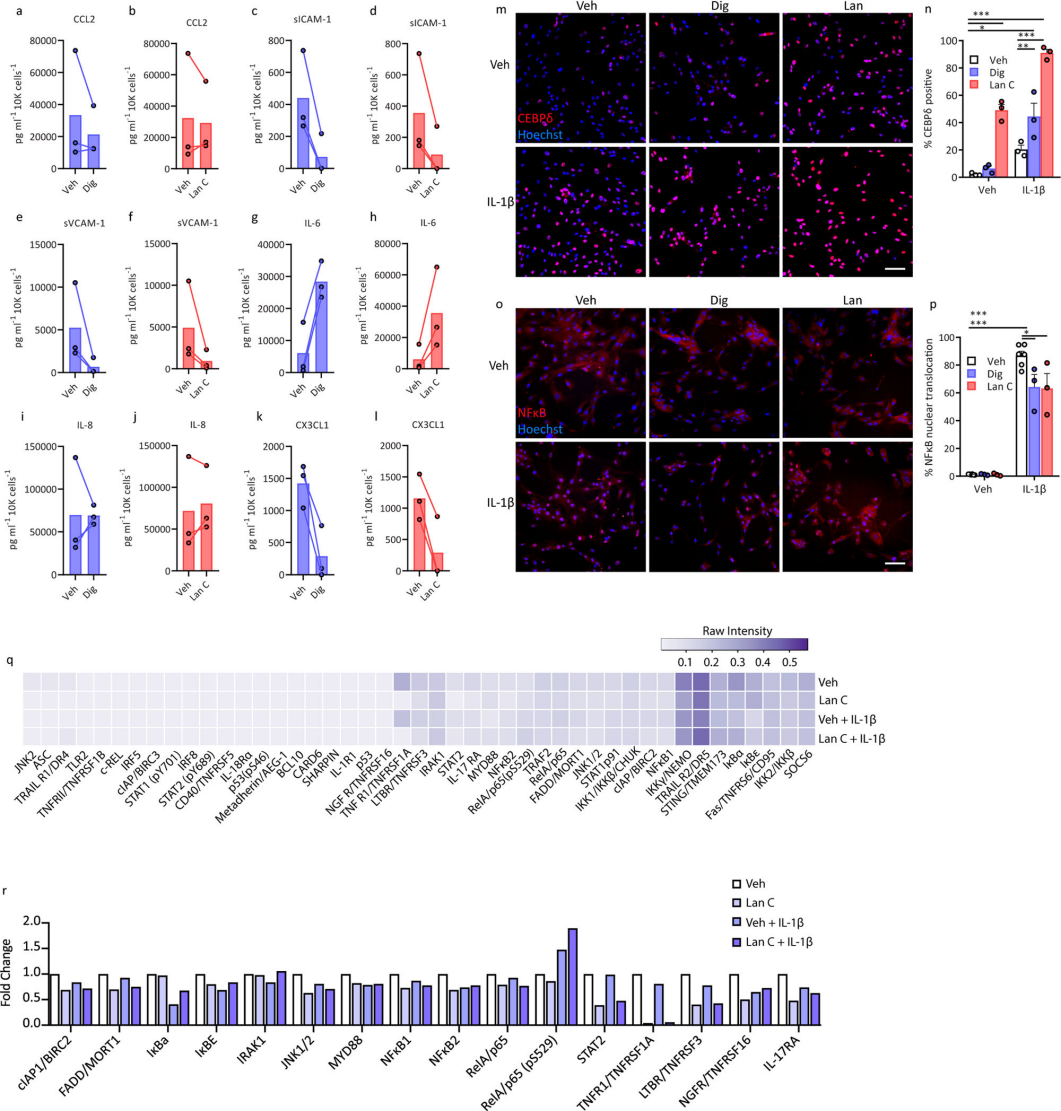
Cardiac glycosides digoxin and lanatoside C modify inflammation-dependent transcriptional responses in pericytes. **a**–**l** Conditioned media from pericytes after 24 h of vehicle, digoxin (blue bars) or lanatoside C (red bars) (125 nM points selected from concentration curves in Fig. S2) pre-treatment prior to stimulation with IL1β (0.05 ng mL^−1^) for 24 h (*n* = 3, cases used: E206, E213, E214). **m**–**p** Pericytes pre-treated with digoxin (100 nM) or lanatoside C (1 µM) for 24 h were treated with IL-1β (0.05 ng mL^−1^) for 1 h, and stained for CEBPδ or NFκB, (representative images (**m** and **o**, scale = 100 µm), (**n**) quantification of CEBPδ percent positive cells, and (**p**) percent NFκB nuclear translocation were compared to control conditions. Two-way ANOVA with Tukey’s multiple comparison test, mean ± SEM.***p < 0.001, **p < 0.01, *p < 0.05, (*n* = 3, cases used: E204, E206, E208). **q** Pericytes were pre-treated with vehicle or lanatoside C (1 µM) for 24 h, then treated with vehicle or IL-1β (0.05 ng mL^−1^) for 30 min. Lysates (500 µg per sample) were analysed using the human NFκB array kit. Average intensity values from duplicate spots were normalised to the average intensity of the reference spots on each blot (*n* = 1, case used: E213). **r** Select targets from NFκB profiler presented as fold change in intensity from vehicle.

Pericytes express several proteins that facilitate leucocyte extravasation and polarise surrounding immunologically-active cells to pro- or anti-inflammatory phenotypes^42,47^. Concentration-dependent secretion data from pericytes pre-treated with digoxin and lanatoside C were consistent with an inhibitory effect on IL-1β-induced soluble adhesion molecule (sICAM-1 and sVCAM-1) and CX3CL1 secretion, whilst IL-6 was increased and interkeukin-8 (IL-8) displayed no change (Fig. 2a–l, concentration response curves (Fig. S2a)). Because complications often arise with excessive dosage of digoxin, several outputs of pericyte viability following digoxin or lanatoside C treatment were examined to rule out the possibility of the inflammatory-modulating effects being a result of cytotoxicity. While both digoxin and lanatoside C reduced pericyte number, this is likely a reflection of reduced proliferation over the treatment period (Fig. S2b, c), as opposed to cell death, which was largely unchanged (Fig. S2d). Interestingly, a reduction in platelet-derived growth factor receptor β (PDGFRβ) expression was observed in response to digoxin and lanatoside C that was consistent with reduced proliferation at this time point (Fig. S2e). Signalling through the PDGFRβ pathway in pericytes promotes their proliferation^48,49^ suggesting that the anti-proliferative effect of digoxin/lanatoside C may result from this PDGFRβ depletion.

Transcription factor activation is a key component of the inflammatory response, in both negative and positive regulatory pathways. CCAAT/enhancer binding protein delta (CEBPδ) has recently been identified as an anti-inflammatory effector in pericytes, downregulating IL-1β-induced CCL2 and ICAM-1 expression^19^. Conversely, in response to IL-1β, TNFα and LPS, nuclear factor kappa-light-chain-enhancer of activated B cells (NFκB) translocates to the nucleus where it induces transcription of numerous pro-inflammatory transcripts^18,50^. Lanatoside C induced CEBPδ nuclear expression under basal conditions, while both digoxin and lanatoside C increased IL-1β -induced expression (Fig. 2m, n). In contrast, both compounds reduced IL-1β-induced NFκB nuclear translocation (Fig. 2o, p) and TGFβ_1_-induced SMAD2/3 translocation (Fig. S3a, b), with no effect on IFNγ-induced signal transducer and activator of transcription 1 (STAT1) activation (Fig. S3c, d), suggesting a selective element of inflammatory pathway inhibition. Further inspection of NFκB pathway components affected by lantatoside C treatment using pericyte cell lysates revealed a lanatoside C-dependent reduction in cellular inhibitor of apoptosis protein (cIAP), Fas-associated death domain (FADD), c-jun N-terminal kinase (JNK1/2), STAT2, nuclear factor of kappa light polypeptide gene enhancer in B-cells inhibitor epsilon (IκBε), TNF receptor-1 (TNFR1) and lymphotoxin-β receptor (LTBR), that appear independent of IL-1β. While lanatoside C treatment partially reversed the IL-β-induced expression changes in interleukin-1 receptor associated kinase (IRAK), IκBα, while exaggerating IL-β-induced phosphorylation of NFκB/p65 at S529 (Fig. 2q, r) pointing towards multiple pathway targeting. Interestingly, gene expression analysis of pericytes under the above conditions do not show a diminished inflammatory response—quite the opposite. Both digoxin and lanatoside C strongly increased transcripts for *CCL2*, *ICAM1*, *VCAM1*, *IL6*, *IL8*, *CX3CL1* and *CEBPD* under basal and IL-1β conditions (Fig. S4). These data suggest that digoxin and lanatoside C may be acting both up and downstream of transcriptional pathways to modulate inflammatory responses, and might be due to the inhibitory actions of cardiac glycosides on cellular protein synthesis^51^.

**Fig. 3:**
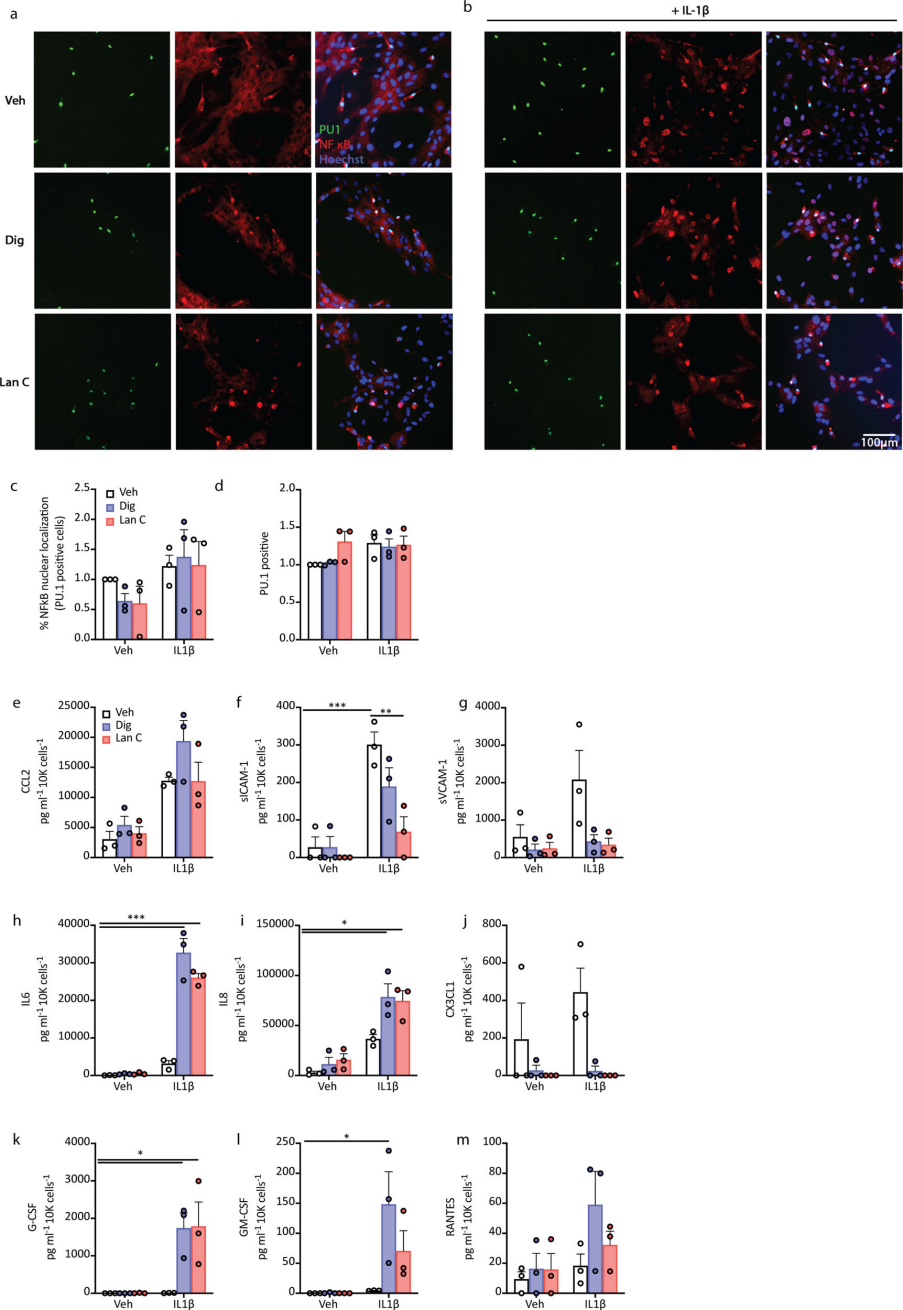
Cardiac glycosides modulate cytokine secretions from mixed glial cultures of microglia and pericytes. **a**, **b** Mixed glial cultures were treated with cardiac glycosides for 24 h prior to stimulation with IL1β for 1 h ((**a**) representative images). Inflammatory response was assessed by NFκB nuclear translocation in PU.1 positive microglia (**c**, **d**). **e**–**m** Conditioned media from mixed glial cultures after 24 h of vehicle (white bars), digoxin (100 nM, blue bats) or lanatoside C (1 μM, red bars) pretreatment prior to stimulation with IL1β (0.05 ng mL^−1^) for 24 h (*n* = 3, case used E215, E216, SS54). Two-way ANOVA with Tukey’s multiple comparison test, mean ± SEM.***p < 0.001, **p < 0.01, *p < 0.05.

In mixed glial cultures, while neither digoxin nor lanatoside C showed any effects on IL-1β-induced NFκB translocation in microglia (Fig. 3a–d), changes in cytokine secretions were consistent with their effects on pericyte cultures (Fig. 3e–m). This is most likely due to the low percentage of microglia present in the predominantly pericyte cultures at early passage (7.3%, ±4.7 PU.1 positive cells). Therefore while we cannot conclude whether the cardiac glycosides tested here alter inflammatory microglial secretions, our data suggests that digoxin and lanatoside C do not modulate IL-1β-induced NFκB translocation in human microglia. This is consistent with previous studies in rodent microglia treated with another cardiac glycoside, ouabain^52^.

### Digoxin and lanatoside C block endothelial inflammatory secretions

Brain endothelia lining the cerebral blood vessels are the first point of contact for therapeutic drugs in the systemic circulation and express numerous active efflux transporters, which complicates CNS drug delivery. Additionally, brain endothelia play an important role in neuroinflammatory responses generated from both the periphery and the brain parenchyma by mediating recruitment, adhesion, and transcellular/paracellular immune cell trafficking across the BBB through expression of cellular adhesion molecules (ICAM-1, VCAM-1) and chemokines (IL-8, CCL2)^53–55^. As p-glycoprotein substrates, an intact BBB would likely exclude digoxin and lanatoside C access to parenchymal brain cells, however reduced expression of drug efflux proteins has been observed in both animal models and in human neuroinflammatory-related diseases^56–58^. Alternatively we propose that targeting inflammatory propagation in endothelia directly may serve to reduce inflammation and activated immune cell entry to the CNS without the need for the drugs to pass the BBB. In order to further assess anti-inflammatory effects of digoxin and lanatoside C, particularly with respect to leucocyte extravasation in the cell type most likely to be exposed in vivo, inflammatory responses of primary adult human brain endothelia were assessed using the aforementioned paradigm described for pericytes.

**Fig. 4:**
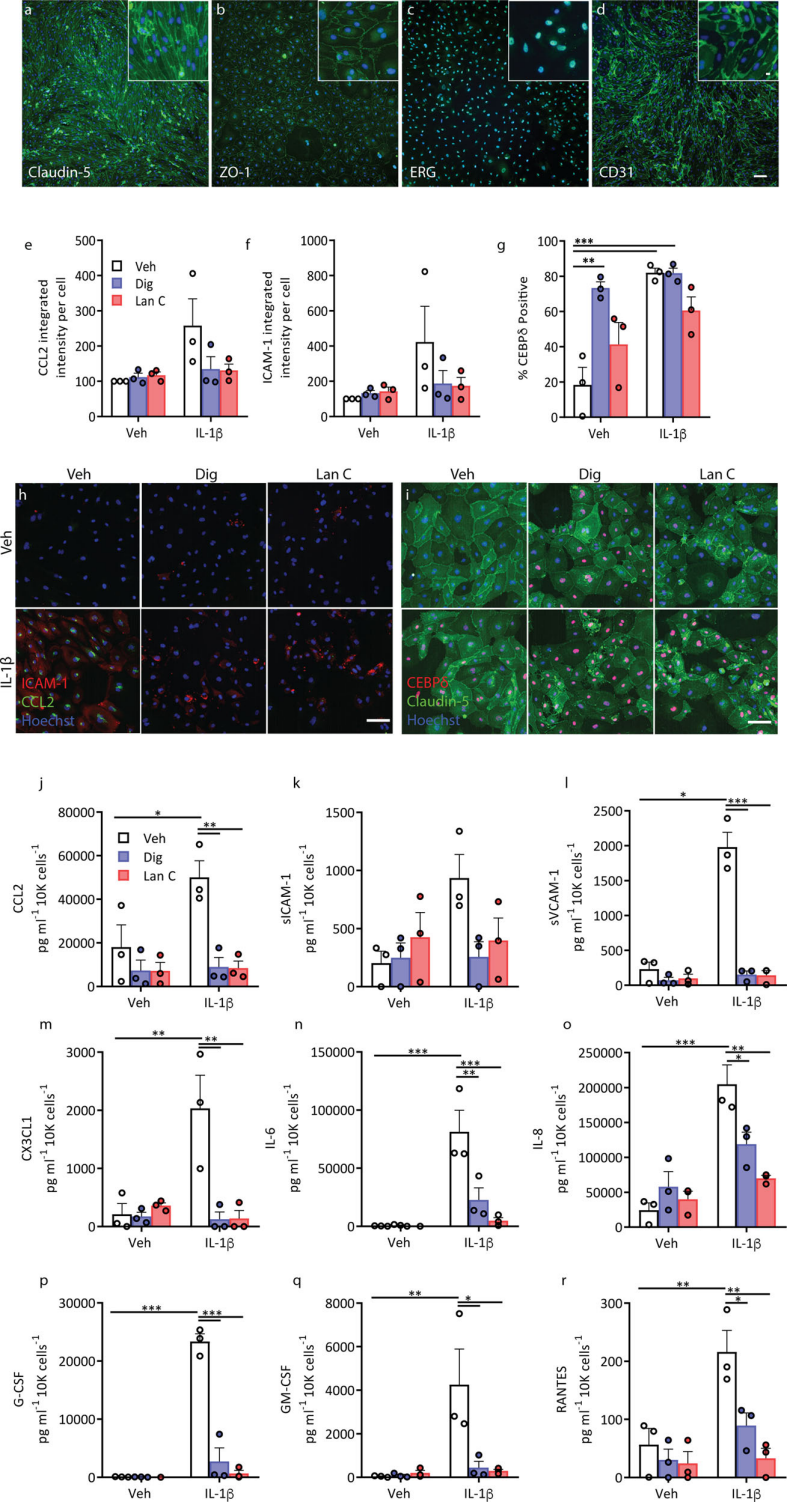
Inflammatory cytokine secretion is blocked by both digoxin and lanatoside C in primary human brain endothelial cells. Endothelial cells derived from human brain tissue express endothelial markers, Claudin-5 (**a**), ZO-1, (**b**), ERG (**c**) and CD31 (**d**). **e**–**g** Human endothelial cells were pretreated with vehicle (white bars), digoxin (100 nM, blue bars) or lanatoside C (1 µM, red bars) for 24 h, followed by 24 h of IL-1β (0.05 ng mL^−1^) treatment. Staining quantification of CCL2 (**e**), ICAM-1 (**f**) and nuclear CEBPδ (**g**), in endothelial cells (*n* = 3, cases used: SS42, SS50, E214). Representative images (**h**, **i**). **j**–**r** Conditioned media was analysed by CBA in endothelial cells treated as above (*n* = 3, cases used: SS42, SS50, E214). Cytokine/chemokine concentration was normalised to total cell counts (Hoechst)). Two-way ANOVA with Tukey’s multiple comparison test mean ± SEM. ***p < 0.001, **p < 0.01, *p < 0.05.

Expression of endothelial markers Claudin-5, ETS-related gene (ERG), zonula occludens-1 (ZO-1) and platelet/endothelial cell adhesion molecule-1 (PECAM/CD31) were confirmed in primary cultures of human brain endothelia in vitro (Fig. 4a–d). While neither digoxin nor lanatoside C significantly altered basal CCL2 or ICAM-1 expression by immunocytochemistry, digoxin significantly increased CEBPδ expression (Fig. 4e–i). Both digoxin and lanatoside C blocked IL-1β-induced secretion of all analytes measured by CBA (CCL2, sICAM-1, sVCAM-1, IL-6, IL-8, CX3CL1, chemokine(C-C motif) ligand 5/regulated on activation, normal T cell expressed and secreted (RANTES), granulocyte colony-stimulating factor (G-CSF) and granulocyte-macrophage colony-stimulating factor (GM-CSF) (Fig. 4j–r).

### Human meninges and ChP as 3D ex vivo models

Leptomeninges envelop the brain and spinal cord and are composed of the arachnoid and pia mater. The subarachnoid space within the leptomeninges is bathed in ChP-derived CSF and plays a crucial role in protecting the CNS. The anatomical relationship of the vascular cells, meninges and ChP through the subarachnoid, Virchow–Robin/perivascular space and the CSF-containing ventricles allows for the exchange of fluid components be it therapeutics or less desired inflammatory constituents^59^. However, the meninges and ChP can also contribute to numerous neurological disorders, due to their intimate interactions and exposure to the peripheral environment respectively through the meningeal lymphatics and the fenestrated blood vessels^30,60–62^. Early immune responses occur in multiple sclerosis, and meningitis in the meninges and ChP as both are sites of immune cell trafficking into the CNS^28,63–65^. Further, inflammatory secretions in the subarachnoid space and in the ventricular space by the ChP can penetrate the brain to alter cerebral functioning, likely as a consequence of glymphatic influx, whereby neurovascular cells including pericytes, endothelia and astrocytes will also be subjected to cytokine presence^14,66^. In order to further investigate potential anti-inflammatory functions of digoxin and lanatoside C, we first established a method allowing for the ex vivo cultures of human leptomeninges and ChP to study inflammation, and potential drug interventions in a culture system more appropriately recapitulating the in vivo microenvironment than can be achieved by standard in vitro dissociated cultures.

**Fig. 5:**
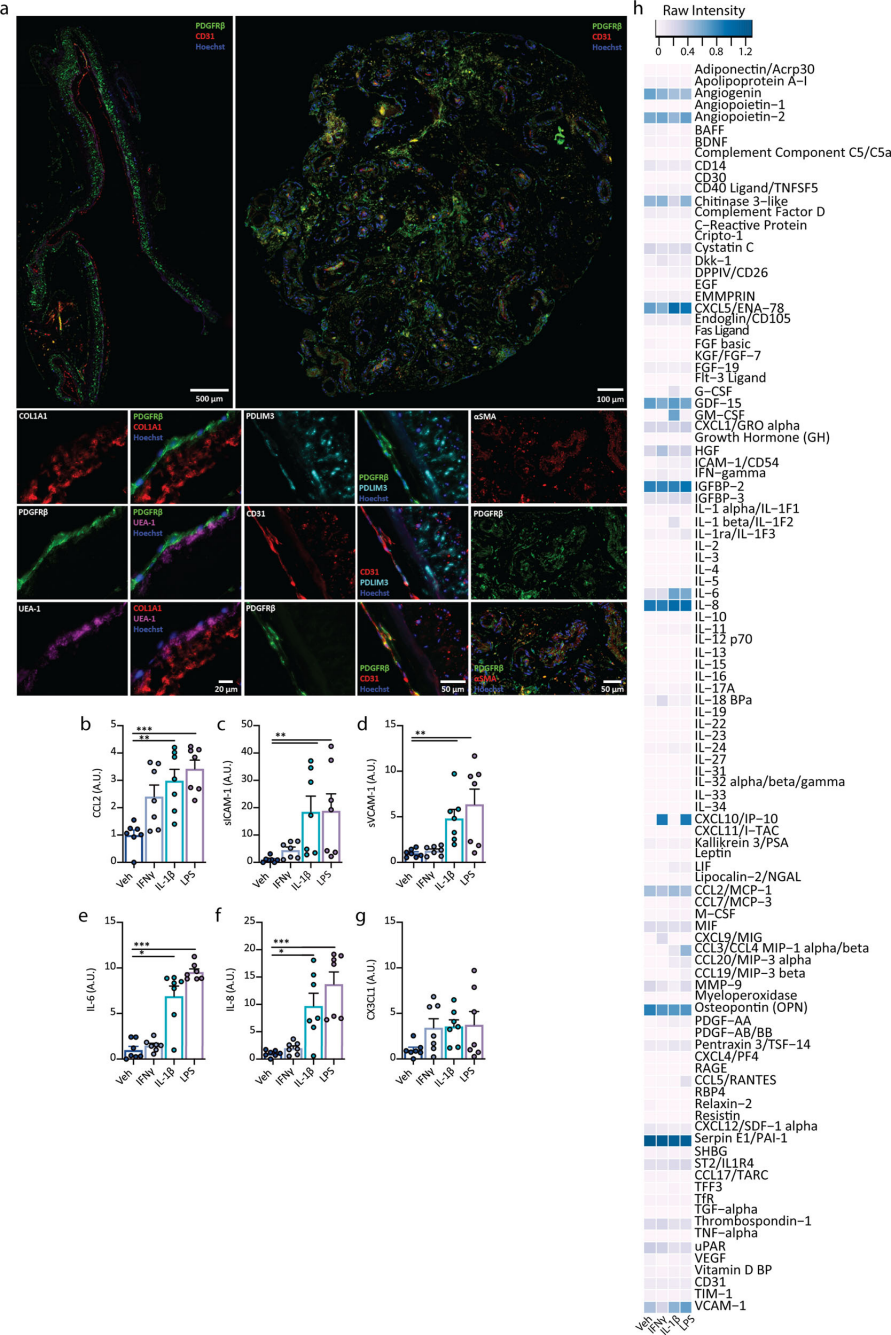
Characterisation of leptomeningeal explant cell types and inflammatory signature. **a** Immunohistochemical staining of LME for vascular cells types including pericytes (PDGFRβ) endothelial cells (CD31), fibroblasts (PDLIM3), smooth muscle cells (αSMA) and extracellular matrix protein deposition (COL1A1). **b**–**g** Secretions from meningeal explants following inflammatory stimulation with vehicle (white bars), IFNγ (blue bars), IL-1β (cyan bars) or LPS (violet bars) were investigated using cytometric bead arrays (*n* = 2 cases used: HC171, AZ129, 3–4 explants per case), values normalised to vehicle condition. One way ANOVA, Dunnett’s multiple comparisons test, mean ± SEM. ***p < 0.001, **p < 0.01, *p < 0.05, raw values Figs. S7 and 8. **h** Proteome profilers were used to detect secretions from human brain meningeal explants (pooled from five explants per condition, one case: H250) after 24 h of inflammatory stimulation with vehicle, IFNγ, IL-1β or LPS (10 ng mL^−1^), raw intensities were normalised to reference spots.

Leptomeningeal explants (LME) derived from drug resistant epilepsy biopsy tissue and post-mortem neurologically normal and disease brains (Fig. S5a–d) were found to be viable in culture, even 3 months after the initial isolation, as determined by live imaging using the ReadyProbes Live/Dead reagents (Fig. S5e–g). Immunohistochemical interrogation of cell specific proteins in LME at experimental endpoints revealed mostly vascular cells, staining positively for pericyte markers PDGFRβ, endothelial cells (CD31, *Ulex europaeus* agglutinin I (UEA-1/lectin)), smooth muscle cells (αSMA), fibroblasts (PDZ and LIM domain protein 3 (PDLIM3)) and extracellular matrix components (collagen, type I, alpha I (COL1A1)) (Fig. 5a)^67^. We also detected cells positive for microglia/macrophage markers Iba-1, HLA-DR and CD68 in both LME and CPE after more than 1 month in culture (Fig. S6). Whilst the inflammatory secretome and cytokine-specific responses of brain pericytes and endothelia have been studied to some extent, significantly less is known regarding this response in the leptomeninges. Secretions from LME in response LPS, IL-1β and IFNγ closely mirrored what we saw in pericytes and endothelia with adhesion molecule and chemokines responsible for immune cell recruitment largely being induced by IL-1β, and LPS, and a lesser response with IFNγ^22^ (Fig. 5b–g, Fig. S7a). Thus, we used cytokine profiler arrays, which allows for simultaneous detection of 102 different soluble inflammatory mediators in response to the aforementioned immunogenic stimuli (LPS, IL-1β and IFNγ). As a likely reflection of their cell heterogeneity, the LME demonstrated an extensive basal secretion of inflammatory mediators, which was differentially induced by LPS, IL-1β and IFNγ (Fig. 5h and Fig. S8a). Importantly, these mediators had no effect on cell viability (Fig. S5f). Notably basal detection of several immune mediators was observed from the LME, in particular angiogenin, angiopoetin-2, chitinase 3-like, C-X-C motif chemokine 5 (CXCL5), growth/differentiation factor 15 (GDF-15), insulin-like growth factor protein-2 (IGFBP-2), IL-8, osteopontin and high basal levels of serpin E1. The most drastic change in secretion in response to cytokine treatment was interferon γ-induced protein-10 (IP-10/CXCL10), which is induced by both IFNγ and LPS, as well as GM-CSF—by IL-1β, and IL-6 by IFNγ and IL-1β.

**Fig. 6:**
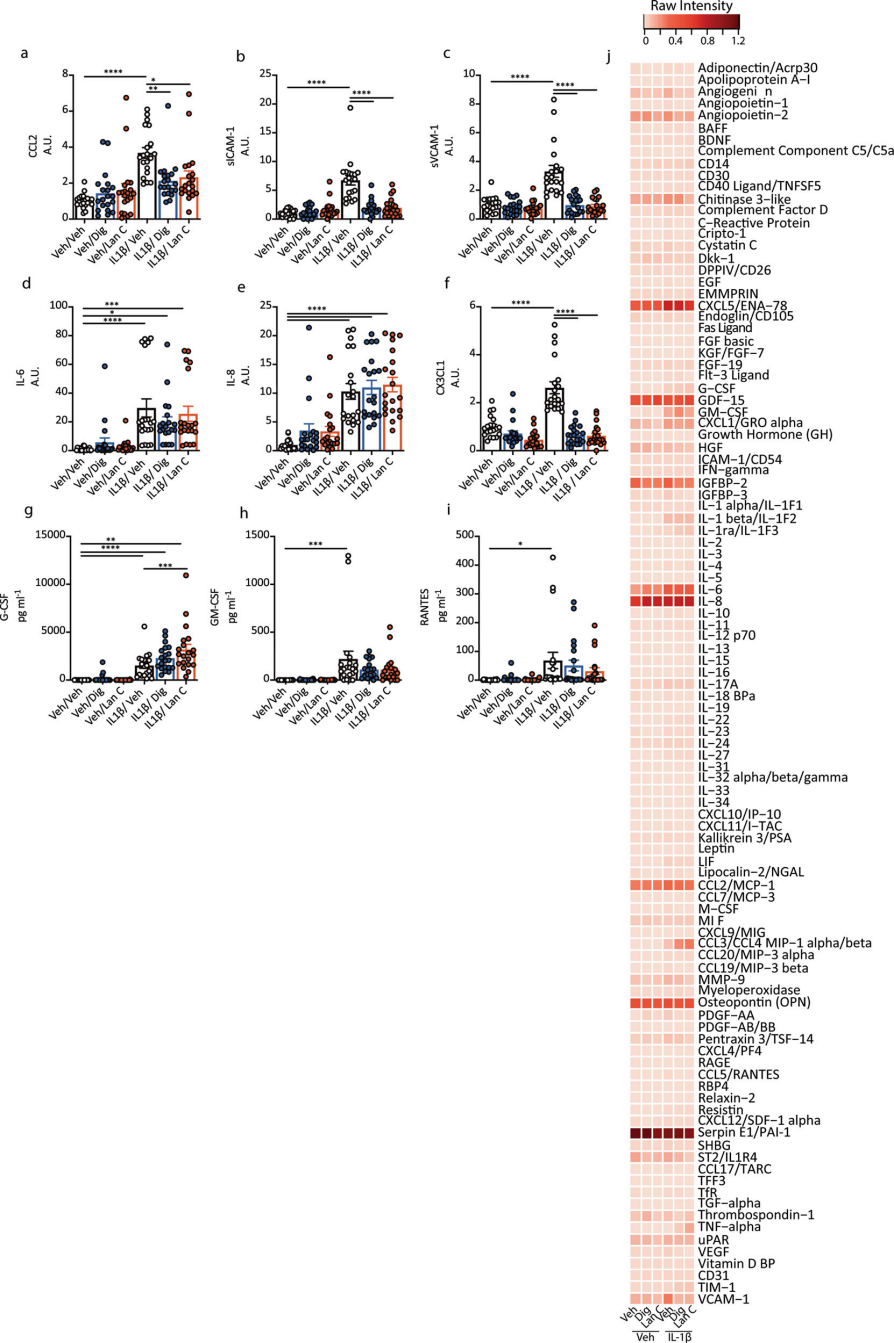
Cardiac glycosides modulate inflammatory responses in leptomeningeal explant cultures. **a**–**i** Meningeal explants were pretreated with vehicle (white bars), digoxin (blue bars) or lanatoside C (red bars) for 24 h then treated vehicle or IL-1β for 24 h. Secretions were measured using CBA (*n* = 3–5 explants per condition, 5 cases: H253, FTD6, HC166, PD85, AZ133). Secretions in pg mL^−1^ were normalised to vehicle for each case-arbitrary units (A.U.) except for G-CSF, GM-CSF and RANTES where cytokines were below the detection level in vehicle conditions. Two-way ANOVA with Tukey’s multiple comparison test, mean ± SEM. ****p < 0.0001, ***p < 0.001, **p < 0.01, *p < 0.05. **j** Proteome profilers were used to characterise LME secretions in response to vehicle or IL-1β (10 ng mL^−1^) with either digoxin or lanatoside C pre-treatment (10 µM). Average intensity values from proteome profilers were normalised to the reference spots on each blot (heatmap is average of *n* = 3 cases used: FTD6, HC166, PD85, each pooled from 3–4 explants per case).

We sought to investigate whether digoxin and lanatoside C could reduce this pro-inflammatory response. As was observed for pericytes and endothelia, digoxin and lanatoside C reduced IL-1β-induced inflammatory mediators in the leptomeninges including CCL2, sICAM-1, sVCAM-1 and CX3CL1 (Fig. 6a–i and Fig. S7b). Using the proteome profilers to interrogate the extent of inhibition by digoxin or lanatoside C we found that both drugs decreased IL-1β–induced secretion of G-CSF, GM-CSF, C-X-C motif chemokine 11 (CXCL11), platelet-derived growth factor AA (PDGF-AA) and sVCAM-1, and they increased secretion of IL-1β dependent IL-6, macrophage inflammatory protein 1-alpha (MIP1α) and TNFα consistently across three LME cases (Fig. 6j and Fig. S7b).

**Fig. 7:**
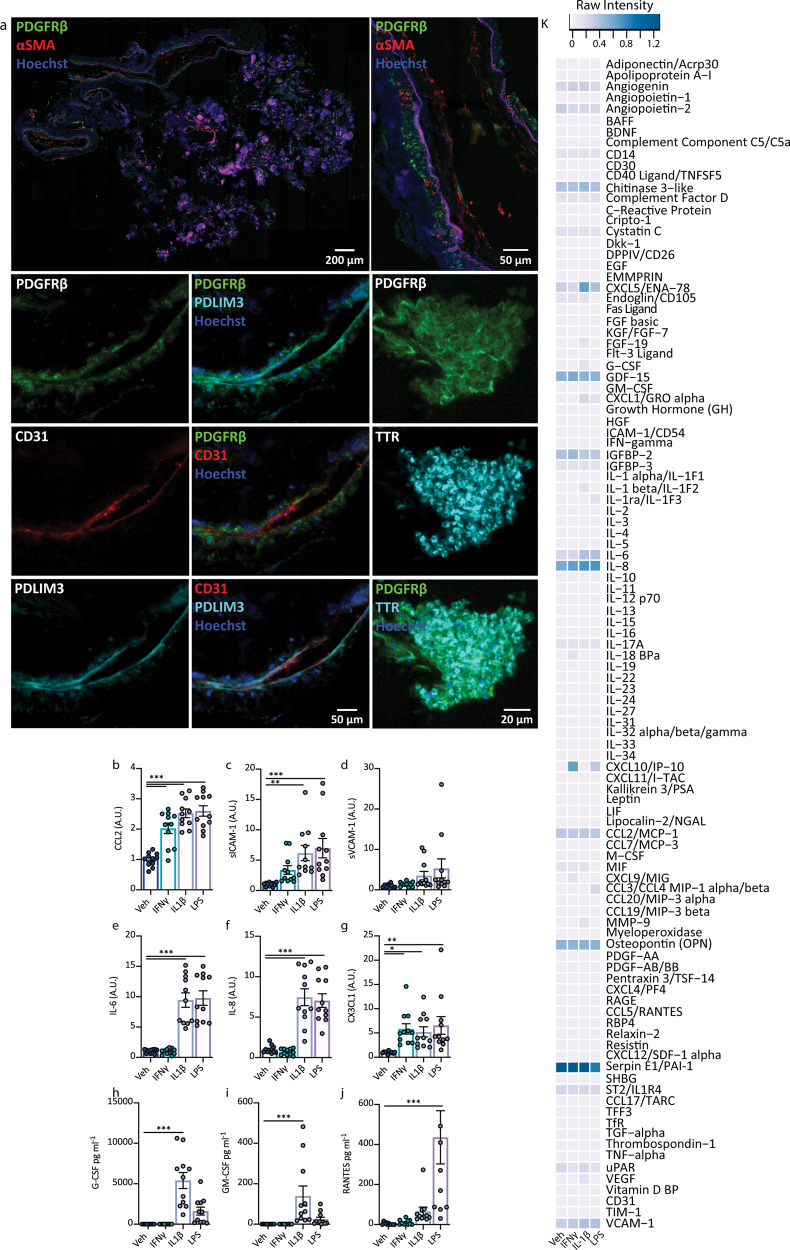
Characterisation of choroid plexus explant cell types and inflammatory signature. **a** Immunohistochemical staining of CPE for vascular cells types including pericytes (PDGFRβ) endothelial cells (CD31), fibroblasts (PDLIM3), smooth muscle cells (αSMA) and epithelial cells (transthyretin (TTR)). **b**–**j** Secretions from CPE following inflammatory stimulation with vehicle (white bars), IFNγ (blue bars), IL-1β (cyan bars) or LPS (violet bars) were investigated using cytometric bead arrays (*n* = 3 cases used: FTD6, HC166, AZ133, three explants per case), values normalised to vehicle condition, raw values Fig. S9. One way ANOVA, Dunnett’s multiple comparisons test, mean ± SEM.***p < 0.001, **p < 0.01, *p < 0.05. **k** Proteome profilers were used to detect secretions from human brain choroid plexus explants (average of *n* = 2 cases used: FTD6, HC166, each pooled from threee explants per condition) after 24 h of inflammatory stimulation with vehicle, IFNγ, IL-1β or LPS (10 ng mL^−1^), raw intensities were normalised to reference spots.

**Fig. 8:**
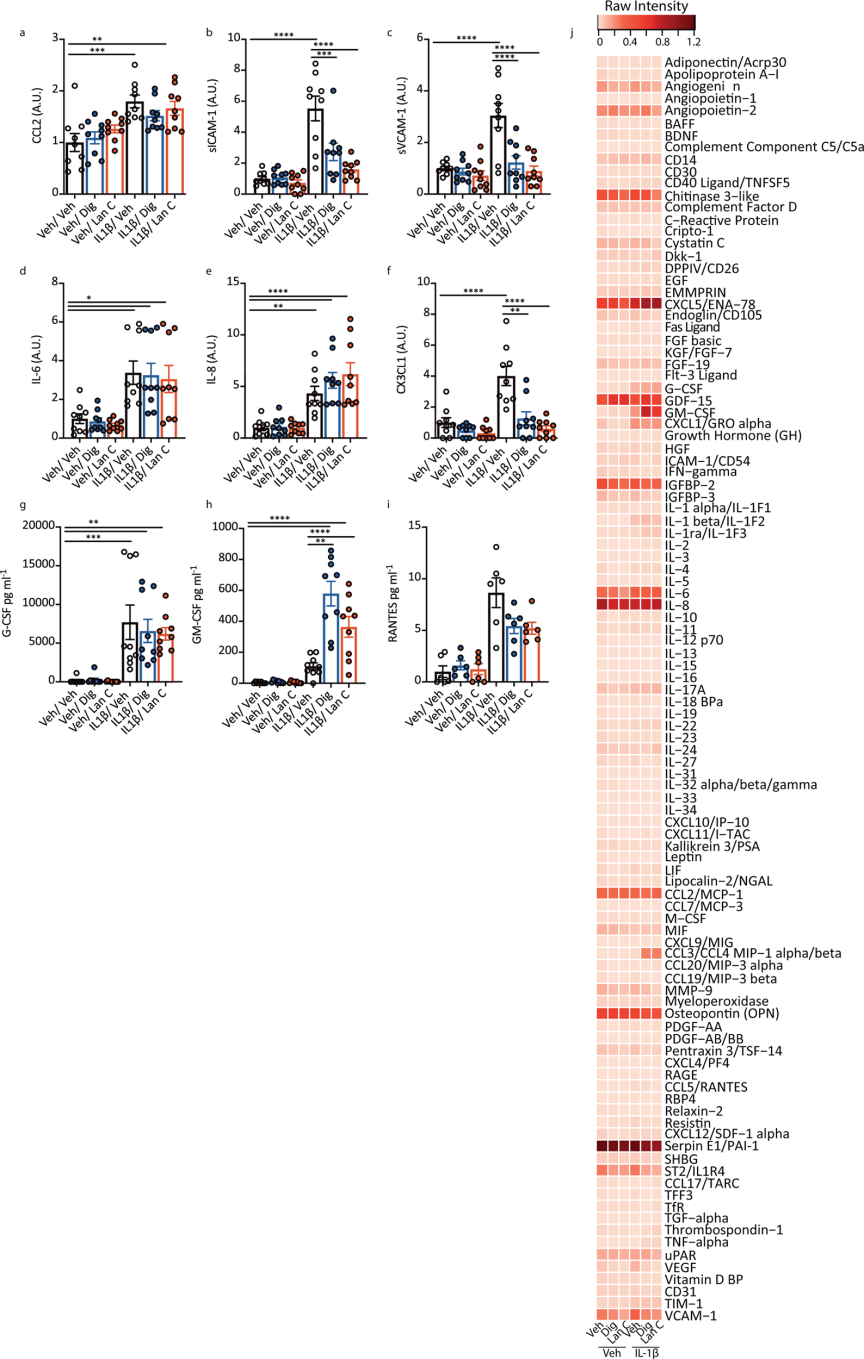
Cardiac glycosides modulate inflammatory responses in choroid plexus explant cultures. **a**–**i** Choroid plexus explants were treated with vehicle (white bars), digoxin (blue bars) or lanatoside C (red bars) (both 10 µM) for 24 h followed by IL1β (10 ng mL^−1^) for 24 h. Secretions were measured using CBA (*n* = 3 (*n* = 2 for RANTES) cases used: HC166, HC171, PD85, three explants per case) ****p < 0.0001, ***p < 0.001, **p < 0.01, *p < 0.05. Values normalised to vehicle for each case, except for G-CSF, GM-CSF and RANTES as vehicle condition was under the level of detection, (raw data provided in Supplementary, Fig. S9). **j** Proteome profiler analysis of pooled choroid plexus explant secretions (*n* = 2, cases used FTD6, HC153 pooled explants per case). Data are presented from the average of each case normalised to reference point.

Using the same paradigm in choroid plexus explants (CPE) we first examined the cellular composition and found the highly vascularised tissue to be harbouring similar cell types to the LME, with the addition of transthyretin-positive epithelia (Fig. 7a). Similar to the LME, the CPE secreted a range of cytokines in response to pro-inflammatory stimulation albeit to a lesser extent than the LME (Fig. 7b–k and Figs. S8, S9 and S10). Similar to the LME under basal conditions, the CPE had undetectable levels of G-CSF, and GM-CSF, very low expression of RANTES, sICAM-1, and noticeable expression of chitinase 3-like, GDF-15, IGFBP-2, IL-8, osteopontin and serpin E1. Expectedly CPE cultures responded to cytokine treatment by changing their secretome with a signature similar to that of LME. Digoxin and lanatoside C pre-treatment resulted in reduced IL-1β- responses such as inhibition of sICAM-1, sVCAM-1 and CX3CL1 secretion by CBA (Fig. 8a–i). Proteome profilers of CPE conditioned media in contrast to LME show an increase in IL-1β induced secretion of G-CSF, GM-CSF and CXCL5, but reduced VCAM-1, angiogenin, osteopontin and interleukin 1 receptor-like 1 (ST2) by digoxin and lanatoside C (Fig. 8j).

## Discussion

Neuroinflammation is present in almost every neurological disorder and contributes to disease pathogenesis by precipitating neuronal loss and BBB dysfunction^5,68^. As such, the identification of therapeutic compounds that effectively attenuate neuroinflammation has the potential to be beneficial in a diverse range of neurodegenerative diseases, neuropsychiatric disorders and acute brain injuries. Unfortunately, compounds that were beneficial in preclinical models of neurological disease, including anti-inflammatory therapies, have largely failed to effectively translate to clinical use^69,70^. Whilst the failure of anti-inflammatory interventions may be a function of inappropriate timing in the treatment regime, the lack of translational therapeutics could be equally attributed to the frequent use of rodent models in neurological drug discovery, in which responses may poorly reflect those observed in humans. Failure may also be associated with targeting of general inflammatory pathways irrelevant to neurodegenerative disease, suggesting that targeting distinct CNS neuroinflammation pathways may prove more successful.

To address this issue, we utilised primary human brain pericytes to screen for anti-inflammatory drugs in a library consisting of 1280 FDA approved compounds. These cells have the obvious benefit of being of human origin, therefore negating any species differences which complicate observed responses. Additionally, unlike human microglia or astrocytes, these cells are highly proliferative in vitro, allowing for efficient bulking and bio-banking of these cultures to generate sufficient yields to undertake large screens^71^. Due to its ubiquitous presence in neurological disease, we sought to target neuroinflammation in this screen in particular because together, endothelia and pericytes are the major mediators of leucocyte extravasation during neurodegenerative diseases, particularly in AD and MS, through CCL2-directed chemotaxis and ICAM-1 and VCAM-1-mediated adhesion^55,72–74^. Alternatively, CNS tumours may be targeted by enhancing peripheral immune infiltration, and the associated mechanisms may be interrogated by using this approach^75,76^. Our screening pipeline included both increases and decreases in CCL2 or ICAM-1—identifying drugs that could potentially be pursued for the purpose of immune cell BBB penetration, although this was not within the scope of this study. However, this approach would be equally suited to identify mitogenic enhancers, autophagy regulators, phagocytic inducers or pro-survival drugs, utilising differing stimulation paradigms.

The strength of the current screening approach resides in the fact that compounds underwent several rounds of analysis in primary human brain cells derived from diverse healthy and diseased post mortem and biopsy samples. The drug effects were consistent across different donors, emphasising the capacity of this screening platform as a tool to identify modifiers of human-specific targets taking into account the inherent human variation from the first stages of drug discovery. With recent advancements in the ability to isolate and culture cells from the human brain, in addition to deriving these cells from patient induced pluripotent stem cells, these screening approaches can be expanded to human microglia, astrocytes and even neurons^77–79^.

Whilst the use of non-human or immortalised cell lines has potential issues with such high-throughput screens, so too does the use of in vitro primary cultures themselves. One limitation is cell yield and because pericytes grow better in 96-well microplates compared with 384-well microplates, the throughput is reduced. Hence, we were only able to use one concentration (we chose 10 μM) of each of the FDA approved drugs for the initial screen, meaning that we may have missed drug effects at lower and higher concentrations. Other limitations of this approach are that pericytes in particular utilised here were subjected to several rounds of passaging in order to obtain sufficient yields, for which phenotypic drift can occur^80,81^. It is therefore unclear whether cells accurately reflect the phenotype observed in vivo. Moreover, the inherent complexity of cellular phenotypes in barrier tissues is poorly represented by in vitro monocultures. This was apparent in the diversity in responses to drug treatment between pericytes and endothelia. Digoxin and lanatoside C reduced adhesion molecule secretion yet increased IL-6 and IL-8 in pericytes, while they abolished all inflammatory secretions in endothelia. This difference in drug responsivity may be attributed to cell-type specific isoform expression of the Na^+^/K^+^-ATPase pump, which has been observed in mouse cerebrovascular cells^82^. Furthermore, differences in inflammatory responses between pericytes and endothelia have been documented previously, for example G-CSF and GM-CSF are not significantly induced in pericytes but are secreted by endothelia in response to IL-1β treatment^22^.

In order to analyse the inflammatory-modulating effects of digoxin and lanatoside C in a more complex, multi-cellular model without the complications of dissociated and passaged cells, a protocol to simply and effectively isolate leptomeningeal and ChP explant cultures was used^83^. These explants display excellent viability, they are easily accessible from the post-mortem brain, or from neurosurgical specimens and their ease of handling makes them an attractive model. The advancements in organotypic slice cultures could soon allow for co-culture methods of LME and CPE with human neurons to define cellular interactions in disease conditions^84^. The explants displayed an extensive inflammatory secretome, including cytokines, chemokines and adhesion molecules. Of equal importance, the anti-inflammatory effects observed using in vitro monocultures of pericytes and endothelial cells were largely consistent with our ex vivo leptomeningeal and CPE cultures, suggesting the appropriateness of both model systems in identifying immune modulating compounds. The ability of these compounds to attenuate inflammatory responses in several distinct cell types is promising with the ability to effectively attenuate global neuroinflammation, as this response is not restricted to one particular region. Furthermore, neither digoxin nor lanatoside C completely blocked inflammatory responses, as can be deleterious, with a basal level of inflammatory capabilities important for homoeostasis and defence responses^85^.

The utilisation of an FDA-approved compound library is advantageous as compounds have already undergone safety trials in humans, easing the repurposing of drugs for other disorders. As such, potential information about efficacy of such compounds can often be gleaned from retrospective analysis of patient cohorts taking these compounds for other indications, therefore expensive and timely clinical human safety trials are not necessary. Further, extensive data are already available on their pharmacological properties^46,86,87^. Digoxin and lanatoside C are structurally very similar and well-known for their inhibitory effects on the Na^+^/K^+^-ATPase pump^88^. Both act similarly in cancer cell lines to inhibit proliferation having actions on tumour necrosis factor-related apoptosis-inducing ligand (TRAIL), Src and protein kinase C δ (PKCδ) pathways. Digoxin is approximately three times more potent than lanatoside C, which is consistent with the data presented here^46,89–91^. Current evidence for digoxin and lanatoside C in anti-inflammatory mechanisms while limited, is suggestive of effects on leucocyte infiltration and extravasation through inhibition of cytokine/chemokine secretion and reduced NFκB expression^92^. Although the efficacy of other cardiac glycosides as anti-inflammatory compounds in brain barrier tissues has not been investigated, cardiac glycosides demonstrate a large range of chemical diversity and absorption, distribution, metabolism, elimination and toxicity (ADMET) properties^88^. Nonetheless, we have shown that these compounds can act on multiple inflammatory pathways in brain barrier cells including pericytes, endothelia and in LME and CPE. Therefore, they have strong potential as inflammatory modulating drugs in the context of neuroinflammation. Our studies do not directly address the mode of action of digoxin and lanatoside C as inflammatory modulating drugs although the induction of CEBPδ^19^ and/or the inhibition of nuclear translocation of NFkB may be involved. Other mechanisms might be related to altered cytoarchitecture (e.g. Fig. 4i).

Neurodegenerative diseases are often a result of several diverging dysregulated pathways, including inflammatory responses, protein aggregation, BBB breakdown and inappropriate clearance mechanisms. As such, the identification of multi-target, multi-action drugs (targeting both neuroinflammatory pathways and blood–brain barrier cells) may prove more effective than single target therapeutics^7^. Moreover, accessibility to vascular cells via perivascular spaces, and tissues such as the meninges and ChP through the brain boundary regions and CSF compartments should be considered in drug delivery strategies. Here we demonstrate the utility of repurposed drug screens in human brain cells and identify drugs for use as therapeutic agents in attacking the neurodegenerative cascade in cells and tissues that can be more easily reached. Taken together, our described pipeline represents a promising approach for neuroinflammatory and neurodegenerative drug development.

## Methods

### Study Design

This study was designed to identify chemical compounds that could modulate inflammatory responses in human brain vascular cells. Using post-mortem brain tissue of healthy and disease patients, as well as epilepsy biopsy tissue we screened 1280 FDA-approved compounds against an IL-1β-induced inflammatory response. Candidates identified from the initial screen by CCL2 and ICAM-1 expression in pericytes were forwarded for secretion analysis, and transcription factor activity in pericytes, endothelial cells and mixed glial cultures. Meningeal and CPE were tested as an ex vivo model of neuroinflammation.

### Tissue source

Biopsy human brain tissue was obtained from the Neurological Foundation Human Brain Bank, with informed written consent, from the middle temporal gyrus (MTG) of patients undergoing surgery for drug-resistant epilepsy. Post-mortem leptomeninges were obtained from regions overlying the MTG, and CPE were derived from the lateral ventricle from one hemisphere from neurologically normal individuals, or those with various neurological diseases (Table S1). All brain tissue collection and processing protocols were approved by the Northern Regional Ethics Committee, (AKL/88/025/AM09 New Zealand) for biopsy tissue, and the University of Auckland Human Participants Ethics Committee (Ref no. 011654, New Zealand) for the post-mortem brain tissue. All methods were carried out in accordance with the approved guidelines.

### Primary human brain mixed glial cultures

Biopsy human brain tissue was obtained from the MTG of patients with drug-resistant epilepsy and mixed glial cultures, containing microglia, astrocytes and pericytes, were generated as described previously^78^. Mixed glial cultures were maintained in complete media (DMEM/F12 with 10% FBS and 1% PSG (penicillin 100 U/mL, streptomycin 100 µg/mL, L-glutamine 0.29 mg/mL)) at 37 °C with 5% CO_2_ until confluent. Flasks were trypsinized with 0.25% Trypsin—1 mM EDTA and scraped to detach firmly adherent microglia. Viable cells were counted based on trypan blue exclusion and 5000 cells/well were seeded into 96-well plates in complete media and used for experimentation after 1–3 days. All experiments performed on mixed glial cultures were at passage two.

### Primary human brain pericyte culture

To generate pure pericyte cultures, mixed glial cultures were sub-cultured up to passage four in order to eliminate non-proliferative microglia and astrocytes as described previously^78,93^. Pericyte cultures were maintained in complete media at 37 °C with 5% CO_2_. Viable cells were counted based on trypan blue exclusion and 5000 cells/well were seeded into 96-well plates in complete media and used for experimentation after 1–3 days. All experiments performed on pericytes were at passages four–nine.

### Primary human brain endothelial culture

Primary human endothelial cells were isolated from brain microvessels as described previously^22^. Endothelial cultures were maintained in Endothelia Cell Media (ECM; ScienCell) at 37 °C with 5% CO_2._ Viable cells were counted based on trypan blue exclusion and 10,000 cells/well were seeded into 96-well plates in complete media and used for experimentation when cells had produced a 100% confluent monolayer (typically around 5 days). All experiments performed on endothelia were at passages three-five.

### Compound screening

High-throughput screening to identify compounds, which modulate inflammatory responses in pericytes was performed using the Prestwick Chemical Library (Prestwick Chemical, France) containing 1280 small molecules of FDA approved drugs. Controls included vehicle for compounds (DMSO), TGFβ_1_ (1 or 10 ng mL^−1^), vehicle for IL-1β (0.01% BSA in PBS) or media alone. Pericytes were pre-treated with compounds (or controls) in duplicate at 10 µM for 24 h before treatment with IL-1β (0.05 ng mL^−1^) for another 24 h. At end-point cells were fixed and immunostained for CCL2 and ICAM-1 and nuclei were counterstained with Hoechst 33258 (as detailed below). Cells were imaged using the ImageXpress® Micro XLS automated fluorescent microscope and total Hoechst positive cell counts and the integrated intensity per cell of CCL2 and ICAM-1 was quantified as described previously^18^.

Criteria for advancement from the primary screen to the secondary screen were changes in intensity ± 20% over vehicle + IL-1β values for CCL2 and/or ICAM1 with <15% well variability, and <50% cell loss. The secondary screen was done in triplicate wells, and compounds were moved to the tertiary concentration response stage if meeting cut-off criteria of 20% change over vehicle for IL-1β conditions in either CCL2 or ICAM-1, and <50% cell loss with standard deviations of <15%.

### Immunocytochemistry and high content image analysis

At end-point cells were fixed in 4% paraformaldehyde for 15 min and washed/permeabilized with phosphate buffered saline (PBS) with 0.2% Triton X-100™ (PBS-T). Cells were incubated with primary antibodies diluted in goat immunobuffer (1% goat or donkey serum, 0.2% Triton X-100™ and 0.04% thiomersal in PBS) overnight at 4 °C, (dilutions of antibodies are listed in Table S2). Cells were washed again in PBS-T and incubated with fluorescently conjugated secondary antibodies for 2 h at room temperature. Nuclei were counterstained by a 15 min incubation with 20 μM Hoechst 33258 (Sigma). Quantitative analysis of intensity measures and scoring of positively stained cells were performed using the Cell Scoring, Multiwavelength Cell Scoring and Nuclear Translocation modules on MetaXpress® software (Molecular Devices) as previously described^49^.

### Cytometric bead array

Conditioned media was collected from samples and centrifuged at 160 × *g* for 5 min to collect possible cells and debris. The supernatant was obtained and stored at −20 °C. Analyte concentrations were measured by CBA (BD Biosciences, CA, USA) as described previously^94^. CBA samples were run on an Accuri C6 flow-cytometer (BD Biosciences, CA, USA). Data were analysed using FCAP-array software (version 3.1) (BD Biosciences, CA, USA) to convert fluorescent intensity values to concentrations utilising a 11-point standard curve (0–10,000 pg/mL). Concentrations were normalised to total cells as determined by Hoechst positive counts for monolayer cells.

### Human NFκB pathway array kit

Human pericytes were seeded into 10 cm dishes and allowed to reach confluency. Pericytes were treated with DMSO (0.01%) or lanatoside C (0.1 µM) for 24 h then treated with IL-1β (0.05 ng mL^−1^) for 30 min. Cells were lysed on ice with kit lysis buffer containing protease inhibitor tablets (Roche), scraped into pre-chilled tubes and incubated on ice for 30 min with intermittent vortexing. Lysates were centrifuged at 14,000 × *g* for 15 min at 4 °C, and protein concentration of the supernatant was quantified using the DC protein quantification kit (Bio-Rad). Samples were stored at −80 °C until array analysis. Arrays were carried out following the manufacturer’s instructions, with 500 µg of protein from each sample used for analysis. Membranes were imaged using the LI-COR Odyssey® FC imaging system (LI-COR Biosciences). Intensity of individual spots was determined using Image Studio™ Lite and values were normalised to the average of the six reference spots on each blot.

### Quantitative polymerase-chain reaction (PCR)

RNA was extracted using the RNAqueous® micro-total RNA isolation kit (Ambion (CA), Life Technologies). cDNA was made from 1.5 μg DNase-1 (Promega)-treated RNA using the Superscript III first-strand synthesis kits (Invitrogen). qRT-PCR was performed using Platinum SYBR Green qPCR SuperMix-UDG with Rox kit (Invitrogen). The level of gene expression was normalised to glyceraldehyde-3-phosphate dehydrogenase (GAPDH) at time zero or untreated conditions using the ΔCt method^95^. The list of primers used is included in Table S3.

### Cytokine profiler

Conditioned media from 3–6 LME was pooled, centrifuged at 160 × *g* for 5 min, and the supernatant was collected and stored at −20 °C. Secretome analysis of the clarified media was performed using the Proteome Profiler™ Human XL Cytokine Array Kit (R&D Systems), as per the manufacturer’s instructions. Chemiluminescent detection of the membranes was performed on the LI-COR Odyssey® FC (LI-COR Biosciences). Intensity of individual spots was determined using Image Studio™ Lite and values were normalised to the average of the six reference spots on each blot.

### EdU proliferation assay

Proliferation of pericytes was determined by incorporation of the thymine analogue 5-ethynyl-2′-deoxyuridine (EdU) with the Click-iT®Assay Kit (Life Technologies C10340) according to the manufacturer’s instructions. Briefly, EdU (5 µM) was added to cells 24 h prior to endpoint and incubated at 37 °C. Cells were fixed with 4% PFA for 15 min at room temperature, rinsed with 3% BSA in PBS and permeabilized with 0.5% Triton X-100 in PBS for 20 min at room temperature. Cells were washed twice with 3% BSA in PBS and then EdU reaction cocktail was added for 30 min at room temperature protected from light. Cells were then washed once more with 3% BSA in PBS and imaged as described above.

### Primary human leptomeningeal and choroid plexus explant culture

Leptomeninges were removed by gross dissection from the brain overlying the MTG, from autopsy tissue of neurologically normal or pathologically confirmed cases of neurodegenerative disorders (AD, PD, HD and FTD), as previously described^83^. Leptomeningeal tissue was washed in complete media in sterile petri dishes and dissected into pieces ~2 mm^3^. LME were placed in individual wells in 500 μL of complete media in a 24-well plate allowing them to remain in suspension. Media changes were performed twice a week. Explants that failed to alter media acidification, as determined by a phenol red colour change, were deemed non-viable and discarded. Explants were cultured for at least a week before using for experiments to allow equilibration but remained viable for up to 4 months after initial isolation (for exact culture timing see Table S1). For functional studies, individual explants were placed in 100 μL of media within a 96-well plate. Please note that explants grown in low media conditions attach to plastic microplates and fibroblast-like cells migrate out from the explant. However, in this study we cultured the explants in high media conditions where there is minimal attachment to the microplate and little cell migration from the explant. Under both media conditions explants from human meninges and ChP are stable and viable for weeks to months.

### Explant viability and histological processing

Cell death was determined using the ReadyProbes™ cell viability imaging kit (Invitrogen). NucBlue live reagent and NucGreen dead reagent stains were diluted into media (2 drops per mL blue, 1 drop per mL green), and added to cells in culture 30 min prior to endpoint and incubated at 37 °C for 30 min. Z-stacks (200–250 slices, 5 μm step) of explants were then acquired using the ImageXpress high content imaging system (Molecular Devices). Two-dimensional projections were used for analysis of positively labelled nuclei with the Custom Module Editor (image analysis pipeline described in detail in supplementary data (Fig. S5).

Immunohistochemical staining on formalin fixed, paraffin embedded explants, sectioned at 7 µm was done as previously described^96^. Antigen retrieval with TRIS-EDTA (pH 9) was performed, followed by blocking with 10% donkey serum prior to antibody incubation. Sections were incubated with primary antibodies (for dilutions see Table S2) overnight at 4 °C, rinsed in PBS then incubated with secondary antibodies and Hoechst 33258 (SIGMA) for 3 h at room temperature in the dark. Sections were imaged using the 20X objective on the Metasystems V-slide scanning microscope and stitched to generate images of whole explants. Control sections where the primary antibody was omitted showed no immunoreactivity. The control experiments showed that the secondary antibodies did not cross-react with each other. All confocal recordings were done using an FV1000 confocal microscope (Olympus) with a 40X oil immersion lens (NA 1.00).

### Statistics and Reproducibility

All cell culture experiments were performed three separate times with cells from different donors. Data were normalised to vehicle controls were indicated in figure legends. Statistical test were performed using Graphpad Prism software, one-way or two-way ANOVA with Tukey’s *post hoc* analysis. Data are presented as the mean ± SEM with individual data points representing a single case, or in the case of explants, a single explant.

### Reporting Summary

Further information on research design is available in the Nature Research Reporting Summary linked to this article.

## Supplementary information

Supplementary Information

Description of Additional Supplementary Files

Supplementary Data 1

Supplementary Data 2

Supplementary Data 3

Supplementary Data 4

Supplementary Data 5

Supplementary Data 6

Supplementary Data 7

Supplementary Data 8

Supplementary Data 9

Supplementary Data 10

Supplementary Data 11

Supplementary Data 12

Supplementary Data 13

Supplementary Data 14

Supplementary Data 15

Reporting Summary

## Data Availability

Raw data from figures is supplied in Supplementary Data files, all other data are available upon reasonable request to the corresponding author.
