# Supplementary material for: Cardiac glycosides target barrier inflammation of the vasculature, meninges and choroid plexus
Source: Commun Biol. 2021 Feb 26;4:260. doi: 10.1038/s42003-021-01787-x (PMC7910294; doi:10.1038/s42003-021-01787-x)
Supplement: Supplementary file 1 — Supplementary Information [file 42003_2021_1787_MOESM1_ESM.pdf]

Supplementary Material

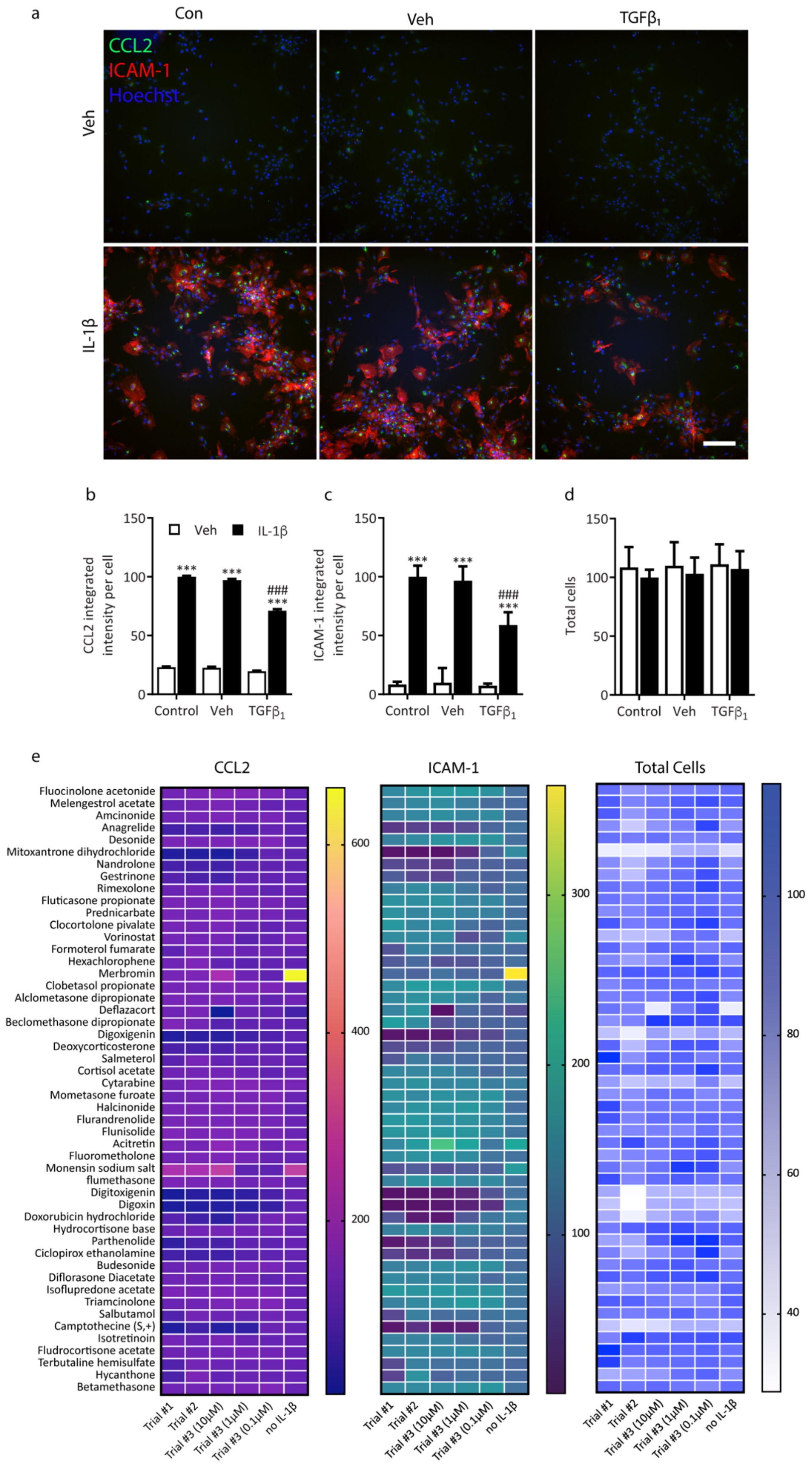
a
Con
Veh
TGFβ1
CCL2
ICAM-1
Hoechst
Veh
IL-1β
b
c
d
150
100
50
0
Veh
IL-1β
CCL2 integrated intensity per cell
ICAM-1 integrated intensity per cell
Total cells
***
###
Control
Veh
TGFβ1
e
CCL2
ICAM-1
Total Cells
Fluocinolone acetonide
Melengestrol acetate
Amcinonide
Anagrelide
Desonide
Mitoxantrone dihydrochloride
Nandrolone
Gestrinone
Rimexolone
Fluticasone propionate
Prednicarbate
Clocortolone pivalate
Vorinostat
Formoterol fumarate
Hexachlorophene
Merbromin
Clobetasol propionate
Alclometasone dipropionate
Deflazacort
Beclomethasone dipropionate
Digoxigenin
Deoxycorticosterone
Salmeterol
Cortisol acetate
Cytarabine
Mometasone furoate
Halcinonide
Flurandrenolide
Flunisolide
Acitretin
Fluorometholone
Monensin sodium salt
flumethasone
Digitoxigenin
Digoxin
Doxorubicin hydrochloride
Hydrocortisone base
Parthenolide
Ciclopirox ethanolamine
Budesonide
Diflorasone Diacetate
Isoflupredone acetate
Triamcinolone
Salbutamol
Camptothecine (S,+)
Isotretinoin
Fludrocortisone acetate
Terbutaline hemisulfate
Hycanthone
Betamethasone
600
400
200
300
200
100
100
80
60
40
Trial #1
Trial #2
Trial #3 (10µM)
Trial #3 (1µM)
Trial #3 (0.1µM)
no IL-1β

Fig. S1. Consistency of hits and controls across drug screen trials in pericytes. (a-d) Validation of controls used for drug screen shows TGFβ_1 (10 ng/mL), a known inhibitor of CCL2 and ICAM-1 expression in pericytes is consistent across all drug plates , ***p< 0.001, compared to control, vehicle treated $^{###}p$< 0.001, compared to control, IL-1β treated. Case used : H239 e) Potential drug hits from three trials (including trial 3, at three concentrations, and one trial with no IL-1β treatment) by immunocytochemical analysis of CCL2, ICAM-1 and total cell counts, normalized to IL-1β treatment for Trial 1, 2, and 3, and no IL-1β trial normalized to vehicle (DMSO) control. Case used: H238.

We also performed Z-Score analyses to determine the degree of plate variability for our pericyte inflammatory screening using CCL2 and ICAM1 (data below). We analyzed the normalized data sets of Vehicle and Vehicle + IL1β for CCL2 (Z-score = 0.43) and ICAM1 (Z-score = 0.15). This analysis shows that CCL2 expression is more consistent across plates than ICAM1, and therefore a superior assay measure. Despite this difference in Z-scores when we correlated (using Pearson's correlation coefficient) the normalized intensity values for CCL2 against ICAM1 for all drug treatments across all plates we observed a very strong positive correlation of r=0.9508, P<0.0001, suggesting that both measures were strongly associated with each other. Although these Z-scores are low, this may reflect the "wild-type" nature of primary cells compared with artificially generated immortalized cell lines. The raw data for this analysis is provided in Dataset 15 raw data for supplementary figure 1.

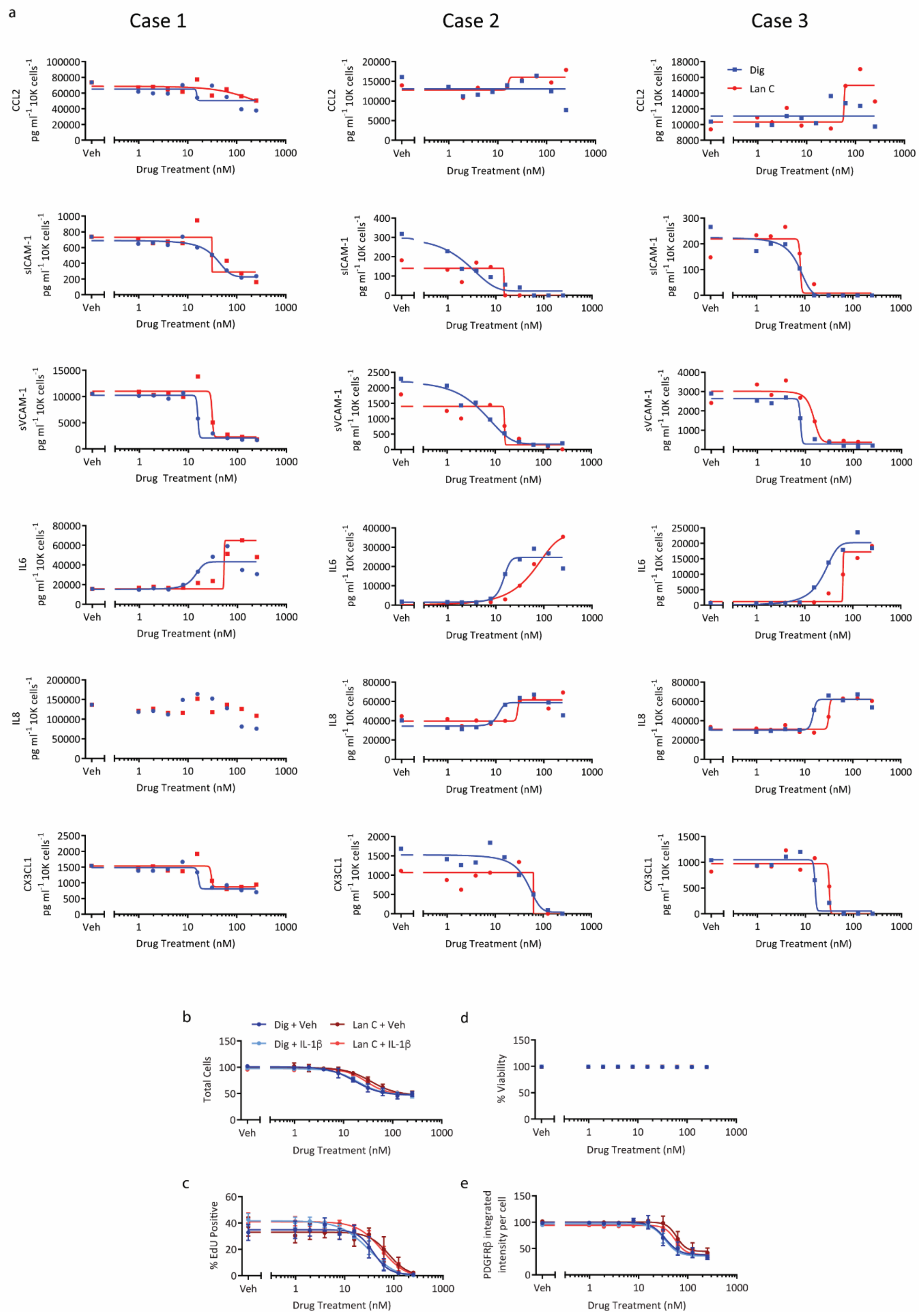

a
Case 1
Case 2
Case 3
Dig
Lan C
CCL2
sICAM-1
sVCAM-1
IL6
IL8
CX3CL1
pg ml^{-1} 10K cells^{-1}
Veh
Drug Treatment (nM)
b
Dig + Veh
Dig + IL-1β
Lan C + Veh
Lan C + IL-1β
Total Cells
c
% EdU Positive
d
% Viability
e
PDGFRβ integrated intensity per cell

Fig. S2. Concentration response of pericytes to digoxin or lanatoside C in the presence of IL-1β. a) Pericytes were pretreated with digoxin or lanatoside for 24 hours prior to IL-1β stimulation. Secretions were quantified with cytometric bead array and normalized to total cells. Case 1 E213, Case 2 E215, Case 3 E216. b) Cells treated as above were assessed for proliferation (EdU), viability (LDH), and PDGFRβ expression. Cases used: E206, E213, E214.

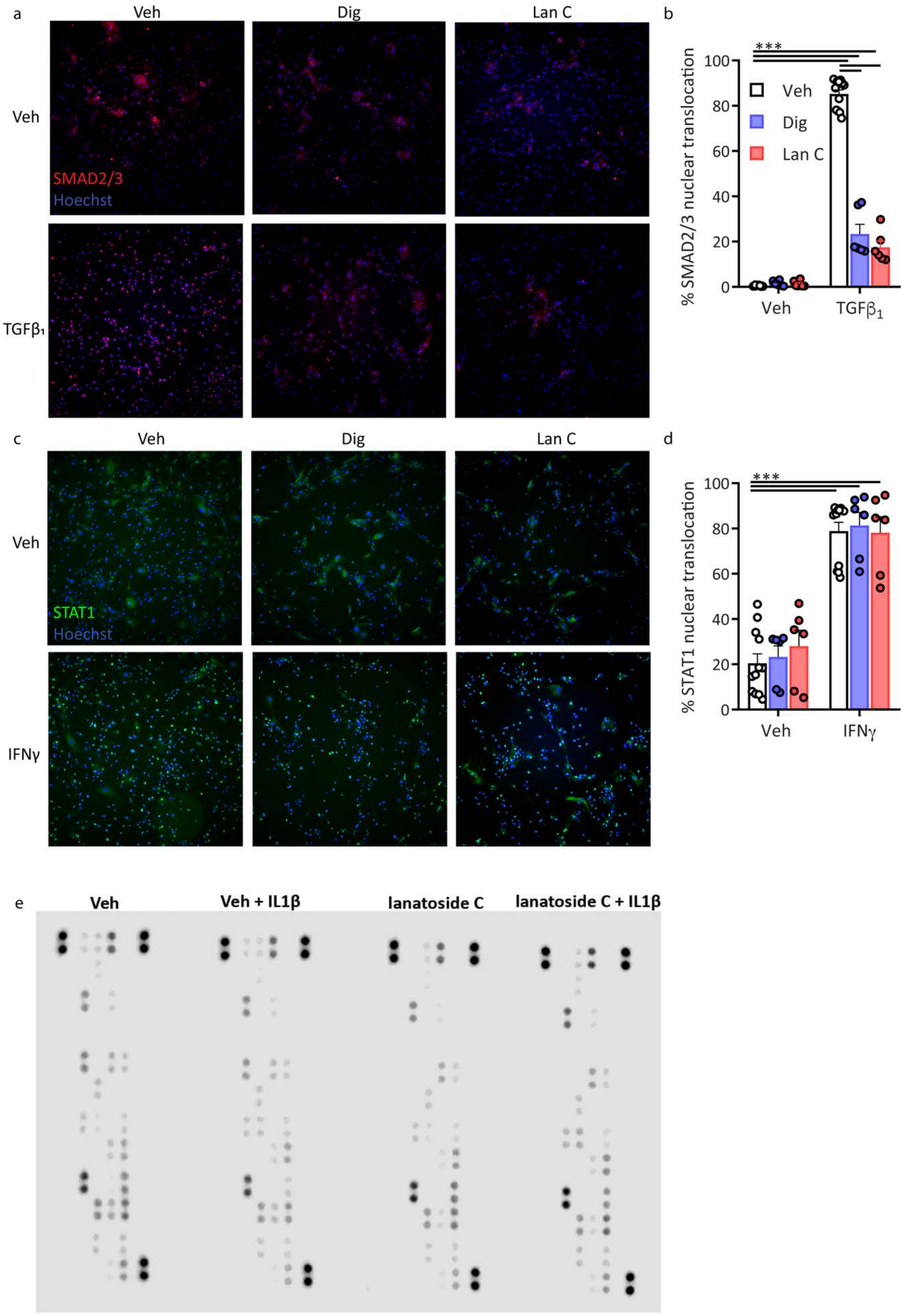
a
Veh
Dig
Lan C
Veh
SMAD2/3
Hoechst
TGFβ₁
b
% SMAD2/3 nuclear translocation
***
Veh
Dig
Lan C
0
20
40
60
80
100
Veh
TGFβ1
c
Veh
Dig
Lan C
Veh
STAT1
Hoechst
IFNγ
d
% STAT1 nuclear translocation
***
0
20
40
60
80
100
Veh
IFNγ
e
Veh
Veh + IL1β
lanatoside C
lanatoside C + IL1β

Fig. S3. Pericytes pre-treated with digoxin (0.1 μM) or lanatoside C (1 μM) for 24 hours were treated with TGFβ$_1$ (1 ng/mL) for one hour and stained for SMAD2/3 (a) or IFNγ (1 ng/mL) for one hour and stained for STAT1 (b). Quantification of nuclear translocation of SMAD 2/3 (c) or STAT1 (d), mean ± S.E.M. ****p*<0.001, (n=3, cases used E203, E204, E213). (e) Blots from NFκB profiler arrays, quantified in Figure 2.

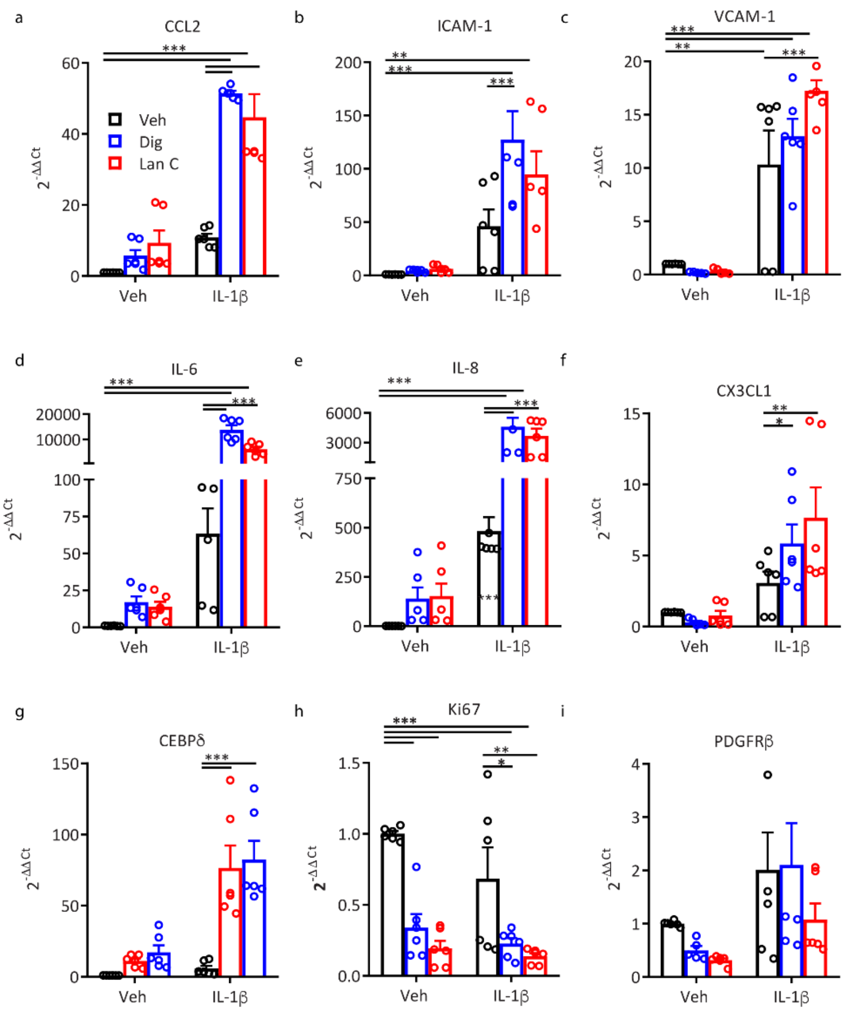

Fig S4. Cardiac glycosides do not block transcriptional activation of IL-1β-dependent inflammatory responses in pericytes. Cells were treated for 24 hours with digoxin (0.1 μM) or lanatoside C (1 μM) then treated with IL-1β for 24 hours. Statistical analysis performed using two-way ANOVA (n=3 cases, 2 experimental replicates), ****p*<0.001, ***p*<0.01, **p*<0.05. Cases used: E206, E213, E214.

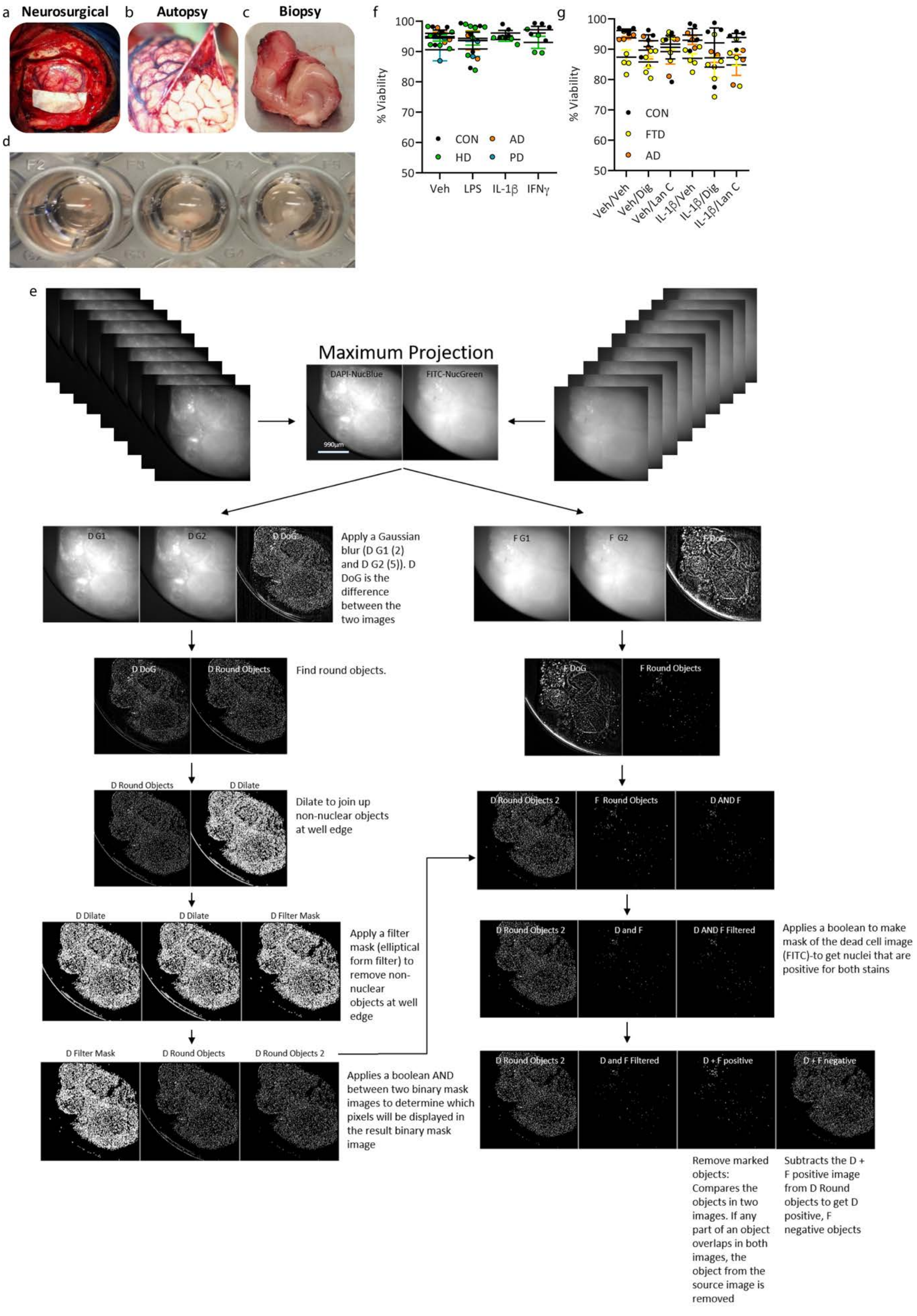

a Neurosurgical
b Autopsy
c Biopsy
d
f
% Viability
CON
AD
HD
PD
Veh
LPS
IL-1β
IFNγ
g
% Viability
CON
FTD
AD
Veh/Veh
Veh/Dig
Veh/Lan C
IL-1β/Veh
IL-1β/Dig
IL-1β/Lan C
e
Maximum Projection
DAPI-NucBlue
FITC-NucGreen
990µm
D G1
D G2
D DoG
Apply a Gaussian blur (D G1 (2) and D G2 (5)). D DoG is the difference between the two images
F G1
F G2
F DoG
D DoG
D Round Objects
Find round objects.
F DoG
F Round Objects
D Round Objects
D Dilate
Dilate to join up non-nuclear objects at well edge
D Round Objects 2
F Round Objects
D AND F
D Dilate
D Dilate
D Filter Mask
Apply a filter mask (elliptical form filter) to remove non-nuclear objects at well edge
D Round Objects 2
D and F
D AND F Filtered
Applies a boolean to make mask of the dead cell image (FITC)-to get nuclei that are positive for both stains
D Filter Mask
D Round Objects
D Round Objects 2
Applies a boolean AND between two binary mask images to determine which pixels will be displayed in the result binary mask image
D Round Objects 2
D and F Filtered
D + F positive
D + F negative
Remove marked objects: Compares the objects in two images. If any part of an object overlaps in both images, the object from the source image is removed
Subtracts the D + F positive image from D Round objects to get D positive, F negative objects

Fig S5. Development of leptomeningeal/choroid plexus explant cultures for ex vivo pharmacological studies. a-c) meninges are sourced from neurosurgeries for the treatment of epilepsy, autopsy or tumour biopsies. Tissue is diced into 2 mm^2 and placed into cell culture dishes with DMEM/F12 (d), 10% FBS, 1% PSG (penicillin 100U/ml, streptomycin 100 µg/ml, L-glutamine 0.29 mg/ml)) at 37 °C with 5% CO_2. e) Viability analysis was performed using the custom module editor function of MetaXpress software to quantify live and dead cells from Z-stacks of explants. f-g) Quantification of explants viability after treatment with cytokines for 24 hours (10 ng/mL), or with pretreatment of digoxin or lanatoside C (10 µM) for 24 hours, followed by IL-1β (10 ng/mL) for 24 hours. Cases used f) H253, HC171, PD85, AZ133, g) H253, FTD6, AZ133.

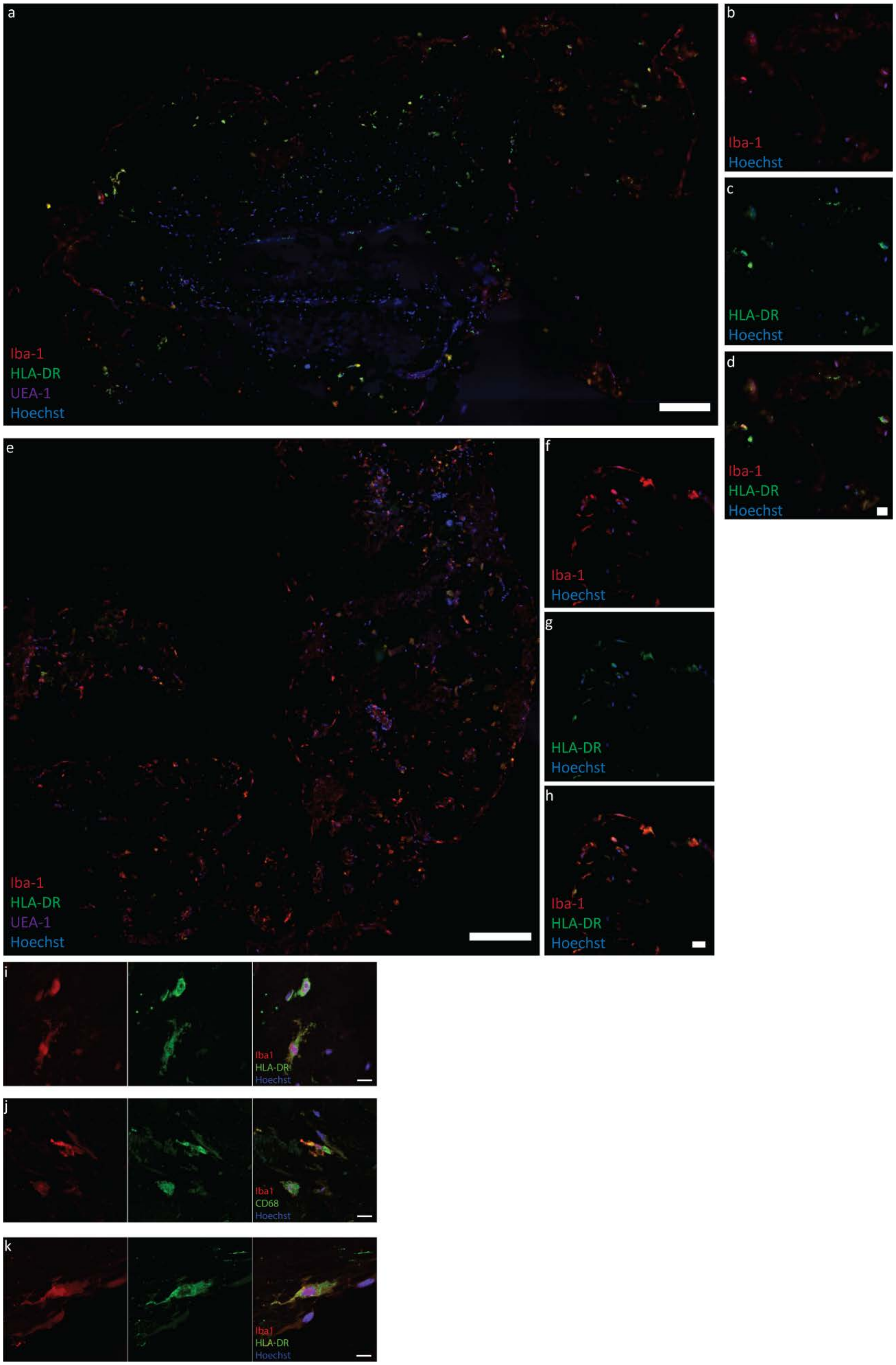

a
Iba-1
HLA-DR
UEA-1
Hoechst
b
Iba-1
Hoechst
c
HLA-DR
Hoechst
d
Iba-1
HLA-DR
Hoechst
e
Iba-1
HLA-DR
UEA-1
Hoechst
f
Iba-1
Hoechst
g
HLA-DR
Hoechst
h
Iba-1
HLA-DR
Hoechst
i
Iba1
HLA-DR
Hoechst
j
Iba1
CD68
Hoechst
k
Iba1
HLA-DR
Hoechst

Fig. S6. Microglia/macrophage presence in leptomeningeal and choroid plexus explants. Immunohistochemical staining of microglial/ macrophage markers in leptomeningeal explants (a-b) (scale 200 μm (a) and 20 μm (b-d), and in choroid plexus explants (e-h) (scale = 200 μm (e) and 20 μm (F-H). i-k) confocal images demonstrating co-expression of Iba-1 with HLA-DR and CD68 (scale = 10 μm).

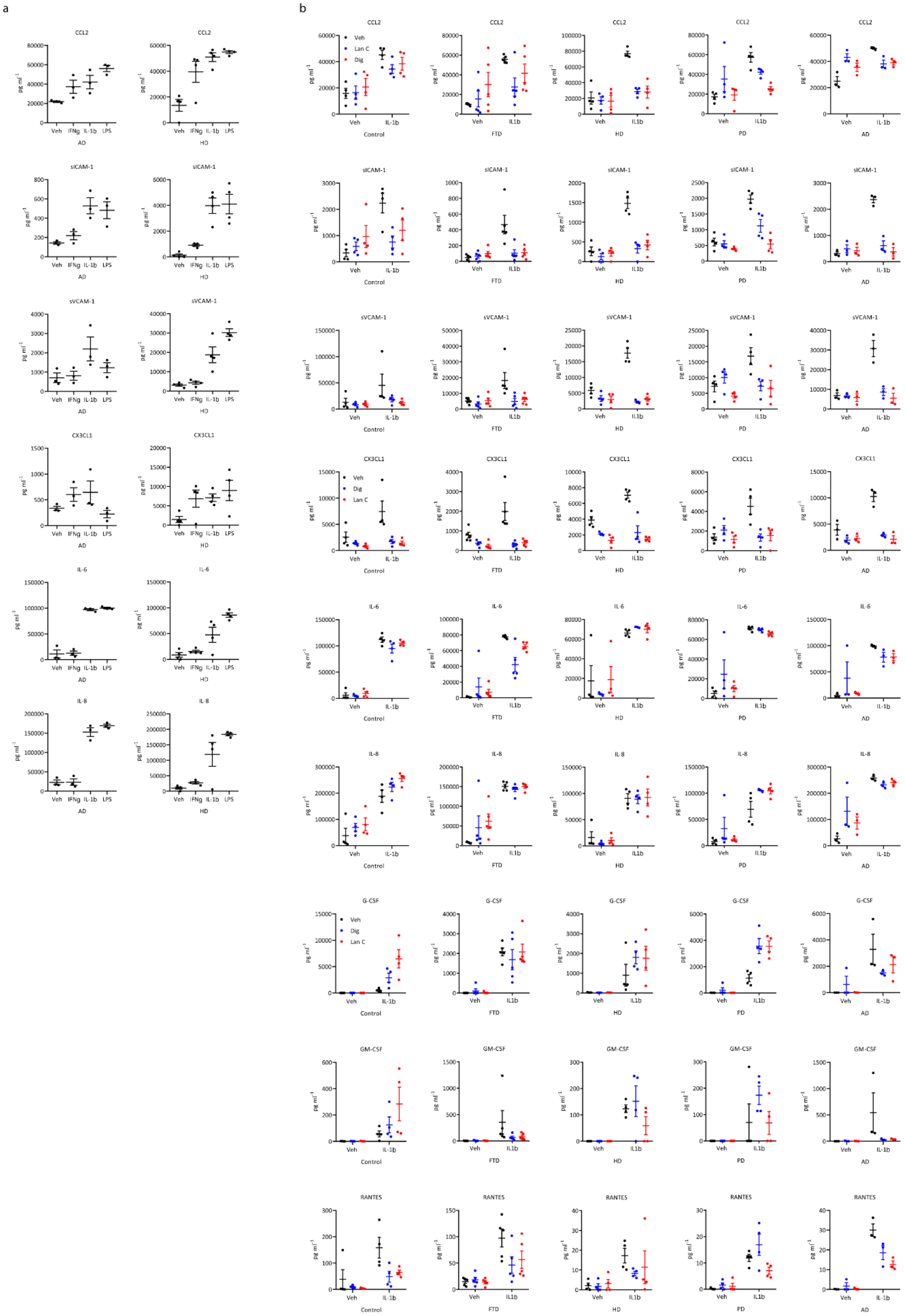
a
b
CCL2
sICAM-1
sVCAM-1
CX3CL1
IL-6
IL-8
G-CSF
GM-CSF
RANTES
Veh
Lan C
Dig
Veh
IFNg
IL-1b
LPS
AD
HD
Control
FTD
PD

Fig S7. a) Raw cytokine concentration from leptomeningeal explants (LME) from two different donors treated with vehicle, IFNγ, IL-1β, or LPS (10 ng/mL), for 24 hours. Cases used H250, AZ133. b) Raw cytokine concentrations from meningeal explants from five different donors LME cases treated with digoxin or lanatoside C (10 μM) for 24 hours then IL-1β (10 ng/mL) for 24 hours. Each point is a single explant. Cases used: H253, FTD6, HC166, PD85, AZ133. (Control- neurologically normal, FTD -frontotemporal dementia, HD -Huntington's disease, PD -Parkinson's disease, AD -Alzheimer's disease). Cases used, H253, FTD6, HC166, PD85, AZ133.

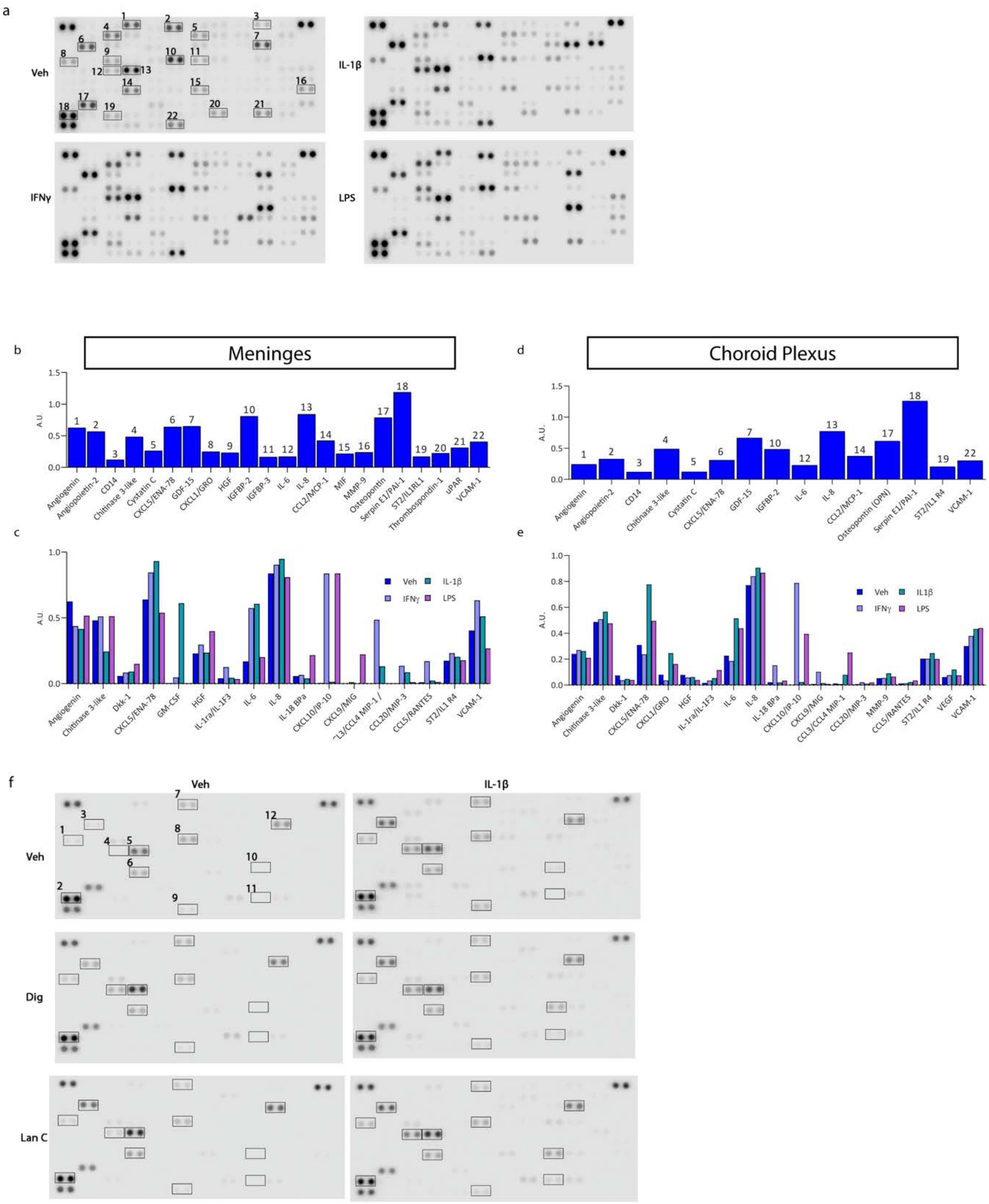

Fig S8. Human cytokine proteome profiler original blots and select hits comparison.

a) Representative blots of LME conditioned media (from Figure 5) exposed to vehicle (Veh), IFNγ, IL-1β, or LPS after 24 hours. Normalized intensity values above an arbitrary threshold under basal conditions in LME (b) or CPE (d). Select secreted proteins that are modified by cytokine treatment in LME (d), or CPE (e). f) Proteome profilers were used to characterise the IL-1β (10 ng/mL) induced changes in secretions from meningeal explants in response with either digoxin or lanatoside pre-treatment (10 μM),

representative blots from one case, pooled secretions from 5 explants. (1-CXCL1, 2- SerpinE1, 3-CXCL5, 4-IL-6, 5-IL-8, 6-CCL2, 7-Ang-2, 8-IGFBP2, 9-VCAM-1, 10-MIP1α, 11-TNFα, 12-GDF-15).

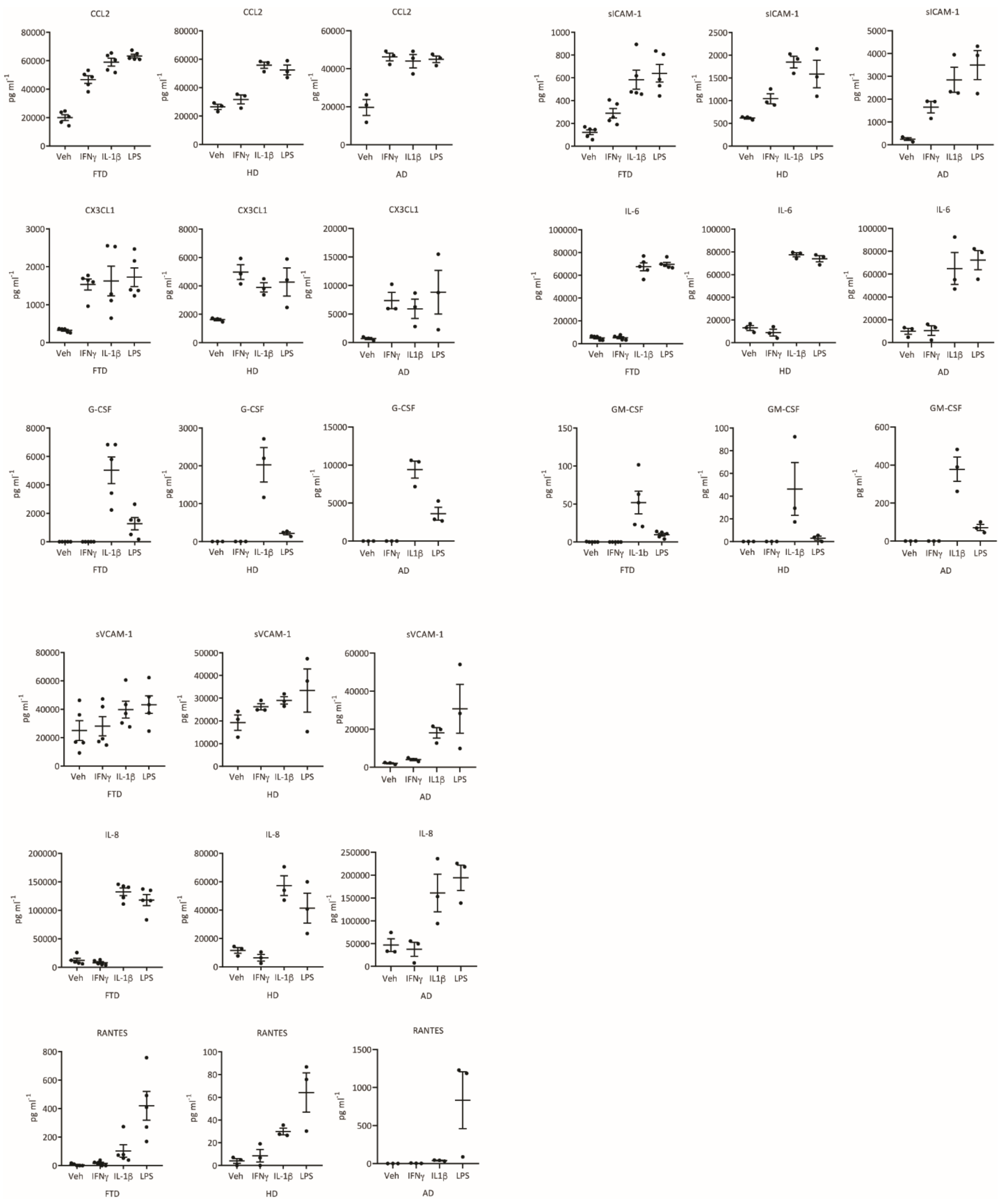

Fig. S9: Raw CBA data from analysis of choroid plexus secretions.

Raw cytokine concentrations from choroid plexus explants from three individual donors (n=3 explants per case) that were treated with vehicle, IFNγ, IL-1β or LPS (10 ng/mL), for 24 hours. Cases used: FTD6, HC166, AZ133.

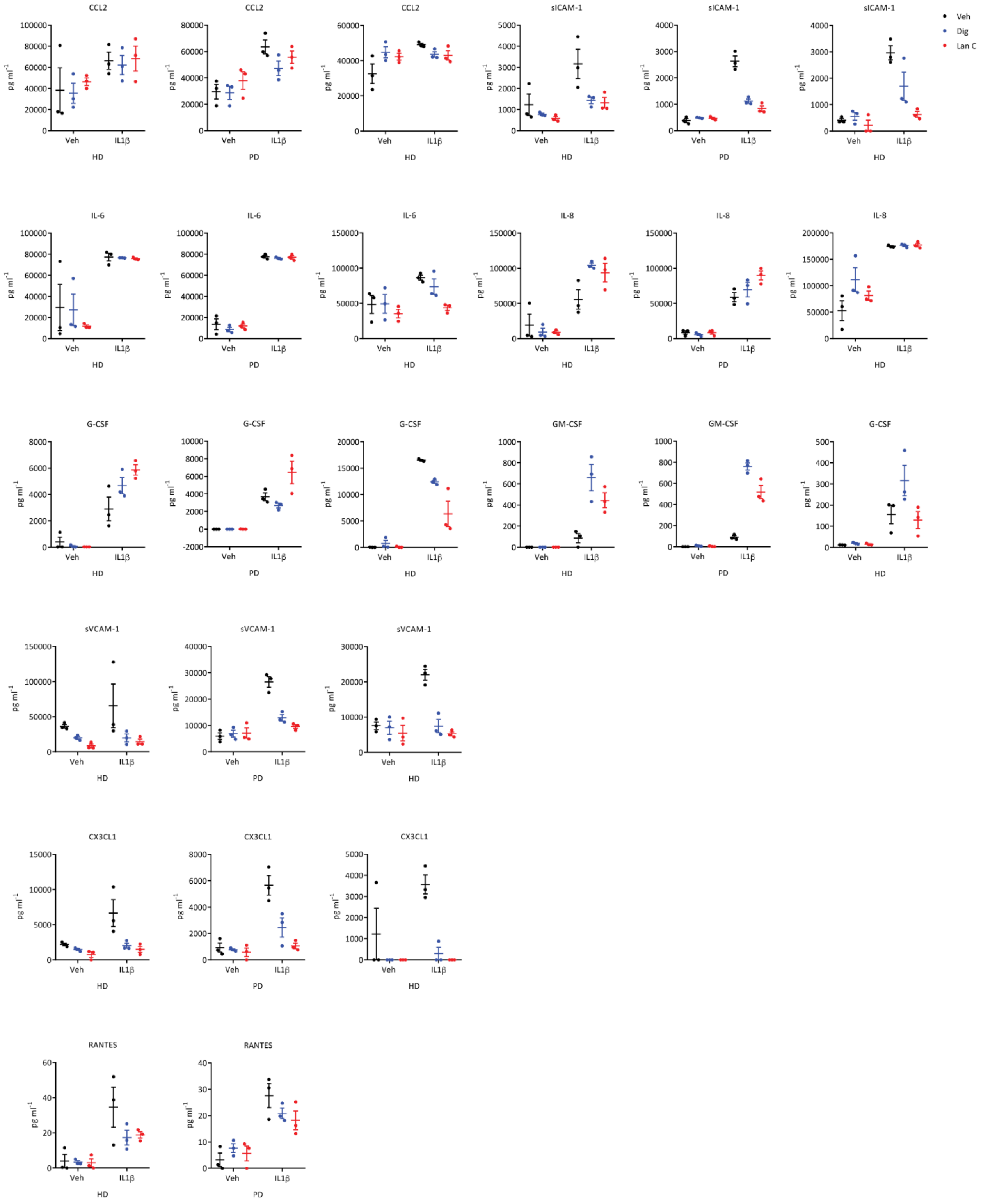

Figure S10: Raw CBA data from analysis of choroid plexus explant secretions (from Figure 8) treated with vehicle, digoxin or lanatoside C (both 10 µM) for 24 hours followed by IL1β (10 ng/mL) for 24 hours.

Table S1. Human tissue sources used in this study.

Case	Tissue source	Confirmed diagnosis	Age	Sex	PMD (hr)	Experiment	Time in culture
E203	MTG	Temporal lobe epilepsy	46	F	<1	ML	Passage 4-9
E204	MTG	Temporal lobe epilepsy	45	F	<1	ML	Passage 4-9
E206	MTG	Temporal lobe epilepsy	29	F	<1	ML	Passage 4-9
E208	MTG	Temporal lobe epilepsy	52	F	<1	ML	Passage 4-9
E211	MTG	Temporal lobe epilepsy	51	F	<1	ML	Passage 4-9
E213	MTG	Temporal lobe epilepsy	23	M	<1	ML	Passage 4-9
E214	MTG	Temporal lobe epilepsy	35	F	<1	ML	Passage 4-9
E215	MTG	Temporal lobe epilepsy	29	F	<1	ML	Passage 4-9
E216	MTG	Temporal lobe epilepsy	38	M	<1	ML	Passage 4-9
SS37	TL	Paediatric epilepsy	9	M	<1	ML	Passage 1
SS42	TL	Paediatric epilepsy	8	M	<1	ML	Passage 1
SS48	TL	Paediatric epilepsy	10	F	<1	ML	Passage 1
SS50	TL	Paediatric epilepsy	3	F	<1	ML	Passage 1
SS54	TL	Paediatric epilepsy	25w	M	<1	ML	Passage 1
H238	MTG	normal	63	F	16	ML, LME	Passage 4-9
H239	MTG	normal	64	M	15.5	ML, LME	Passage 4-9
H250	meninges	normal	93	F	19	LME	7 weeks
H253	meninges	normal	96	M	24	LME, CPE	14 weeks
FTD6	Meninges, ChP	Frontotemporal dementia	76	M	7	LME, CPE	8 weeks
HC166	Meninges, ChP	Huntington's disease	84	F	22.5	LME, CPE	9 weeks
HC171	Meninges, ChP	Huntington's disease	51	M	24	LME, CPE	5 weeks
PD85	meninges/ChP	Parkinson's disease	73	M	4	LME, CPE	9 weeks
AZ129	meninges	Alzheimer's disease	81	M	4.5	LME	9 weeks
AZ133	meninges/ChP	Alzheimer's disease	96	M	21	LME, CPE	5 weeks

MTG-middle temporal gyrus, ML-monolayer cultures, ChP-choroid plexus, LME-leptomeningeal explants, CPE-choroid plexus explants

Table S2. Antibodies and stains used in this study.

**Antibody (species)**	**Catalogue Number (Source)**	**Dilution**
αSMA (mouse)	IS611 (Dako)	1/100 IHC
CCL2 (MCP-1) (Rabbit)	ab-9669 (Abcam)	1/500 ICC
CD31 (mouse)	M0823 (Dako)	1/500 ICC, 1/100 IHC
CD68 (KP1)(mouse)	Ab-955(Abcam)	1/500 IHC
CEBPδ (mouse)	sc-365546 (Santa Cruz)	1/500 ICC
Claudin-5 (rabbit)	ab131259 (Abcam)	1/5000 ICC
COL1A1 (mouse)	sc-293182 (Santa Cruz)	1/50 IHC
HLA-DR (mouse)	M0775 (Dako)	1/1000 IHC
IBA1 (goat)	ab-5076 (Abcam)	1/500 IHC
ICAM-1 (mouse)	sc-107 (Santa Cruz)	1/500 ICC
IL-6 (goat)	AF-206 (R&D Systems)	1/2000 ICC
KCNJ8/Kir6.1 (rabbit)	PA5-56628	1/100 IHC
NFκBp65 (rabbit)	sc-107 (Santa Cruz)	1/500 ICC
PDGFRβ (goat)	AF-385 (R&D Systems)	1/2000 ICC<br>1/200 IHC
PDGFRβ (rabbit)	3169 (Cell Signalling)	1/100 IHC
PDLIM3 (rabbit)	PA5-52099 (Thermo Fisher Scientific)	1/50 IHC
PU.1 (rabbit)	2258 (Cell Signalling)	1/500 ICC
SMAD2/3 (mouse)	sc-133098 (Santa Cruz)	1/500 ICC
STAT1 (rabbit)	14994 (Cell Signalling)	1/500 ICC
Streptavidin Alexa Fluor 647	S21374 (Invitrogen)	2 mg/mL (1/500)
Transthyretin /Pre-albumin (rabbit)	A0002 (Dako)	1/1000
ZO-1 (mouse)	339100 (Invitrogen)	1/500 ICC
Lectin (UEA-1)	L8262 (SIGMA)	1/1000 IHC
Hoechst 33258	SIGMA	1/500 ICC<br>1/10000 IHC

IHC-immunohistochemistry, ICC-immunocytochemistry

Table S3: Primers used for qPCR in this study.

**Gene**		**Sequence**	**Amplicon Length**
CEBPδ	Fw	TTCAGCGCCTACATCGACTC	80 bp
	Rv	TTGAAGAGGTCGGCGAAGAG	
CCL2	Fw	CAGCCAGATGCAATCAATGCC	190 bp
	Rv	TGGAATCCTGAACCCACTTCT	
CX3CL1	Fw	ATTCTTTCCTGAGGCTGGGC	74 bp
	Rv	GGTCTTGGAGGGCAGAGAAC	
GAPDH	Fw	CATGAGAAGTATGACAACAGCCT	113 bp
	Rv	AGTCCTTCCACGATACCAAAGT	
ICAM-1	Fw	GAACCAGAGCCAGGAGACAC	84 bp
	Rv	GAGACCTCTGGCTTCGTCAG	
IL-6	Fw	TTCGGTCCAGTTGCCTTCTC	77 bp
	Rv	TCTTCTCCTGGGGGTACTGG	
IL-8	Fw	CAGAGACAGCAGAGCACACA	70 bp
	Rv	GTGAGATGGTTCCTTCCGGT	
Ki67	Fw	AGCGGAAGCTGGACGCAGAA	79 bp
	Rv	TCCAGGGGTTGGGCCTTTTCCT	
PDGFRb	Fw	CGCAAAGAAAGTGGGCGGCT	101 bp
	Rv	TGCAGGATGGAGCGGATGTGGT	
VCAM-1	Fw	TTGACTTGCAGCACCACAGG	85 bp
	Rv	TCGTCACCTTCCCATTCAGTG	
