# Supplementary material for: Cardiac glycosides target barrier inflammation of the vasculature, meninges and choroid plexus
Source: Commun Biol. 2021 Feb 26;4:260. doi: 10.1038/s42003-021-01787-x (PMC7910294; doi:10.1038/s42003-021-01787-x)
Supplement: Supplementary file 2 — Description of Additional Supplementary Files [file 42003_2021_1787_MOESM2_ESM.pdf]

Description of Additional Supplementary Files

File name: Supplementary Data 1

Description: Fig 1 raw data.xlsx

File name: Supplementary Data 2

Description: Fig 2 raw data.xlsx

File name: Supplementary Data 3

Description: Fig 3 raw data.xlsx

File name: Supplementary Data 4

Description: Fig 4 raw data.xlsx

File name: Supplementary Data 5

Description: Fig 5 and Fig S8 raw data.xlsx

File name: Supplementary Data 6

Description: Fig 6 raw data.xlsx

File name: Supplementary Data 7

Description: Fig 7 raw data.xlsx

File name: Supplementary Data 8

Description: Fig 8 raw data.xlsx

File name: Supplementary Data 9

Description: Fig S1 raw data.xlsx

File name: Supplementary Data 10

Description: Fig S2 raw data.xlsx

File name: Supplementary Data 11

Description: Fig S3 raw data.xlsx

File name: Supplementary Data 12

Description: Fig S4 raw data.xlsx

File name: Supplementary Data 13

Description: Fig S5 raw data.xlsx

File name: Supplementary Data 14

Description: Fig S7-10 raw data.xlsx

File name: Supplementary Data 15

Description: Raw data for Supplementary Figure 1 - Z-score analysis.
