# Supplementary material for: Cardiac glycosides target barrier inflammation of the vasculature, meninges and choroid plexus
Source: Commun Biol. 2021 Feb 26;4:260. doi: 10.1038/s42003-021-01787-x (PMC7910294; doi:10.1038/s42003-021-01787-x)
Supplement: Supplementary file 17 — Supplementary Data 15 [file 42003_2021_1787_MOESM17_ESM.pdf]

Vehicle – CCL2 Intensity	Vehicle + IL1b – CCL2 Intensity	Vehicle – ICAM1 Intensity	Vehicle + IL1b - ICAM1 Intensity
23.47099812	103.1221596	8.884944	104.1636
28.7321512	106.6094128	16.63058	100.9518
25.5229916	94.74541166	10.88392	98.64578
31.88303001	88.39424196	19.38852	90.46519
22.38097446	114.7007015	8.910441	115.0879
22.20597768	106.8288724	7.776651	108.045
16.77714053	102.7606361	5.236389	131.2032
23.44321622	98.85351854	8.182408	98.74832
22.54136959	96.47767104	8.147265	99.83259
24.45630207	95.12734801	8.331453	93.76803
19.67447515	91.05165696	6.993568	85.84629
21.22536147	95.336108	7.10169	93.86295
25.51013506	85.98469682	9.816627	75.29108
25.13464949	116.0790323	11.15463	118.94
25.68836425	93.21345853	10.41555	97.07168
20.65013201	95.21723766	8.156227	100.6159
28.17602719	82.74161621	10.84079	78.69711
23.96825732	94.15209188	8.851324	88.73469
24.89940917	107.2534394	8.768437	102.1136
16.76592433	96.29734519	5.959633	93.30912
21.53121009	103.1536926	7.67293	103.1372
23.64524785	101.0380921	9.5296	103.3502
24.31173067	103.0019418	9.26803	94.58424
62.01747242	97.68416749	106.7276	90.62958
18.06266729	89.41565487	5.948236	82.06792
14.75565912	91.80716302	4.929993	90.40333
16.99059414	98.63846431	5.71396	99.04567
24.06853084	106.3217472	8.90637	105.2632
24.5884612	89.47270549	8.897706	82.24021
39.53123541	105.2803241	12.25676	114.2487
14.94111811	95.50194433	5.321627	93.64402
15.1352896	93.0693762	3.997793	69.91604
23.81860935	104.8822464	8.76285	106.6683
24.83168873	105.2413294	9.703495	101.9382
18.87570444	82.09041612	7.386486	85.05378
18.41225146	93.61913147	7.104876	95.05888
22.25648458	101.6578756	8.510881	100.3714
20.80635695	99.67923455	7.323312	98.08697
14.07102916	96.47270682	4.781258	94.86731
21.05213333	95.09175635	7.580109	96.43263
21.97069928	94.16340789	8.104515	93.76971
20.10882747	97.60637685	7.56939	101.1148
20.11144793	97.25137927	7.020177	96.70219
28.31313632	95.97580731	10.67933	97.53963
24.02378733	115.0009073	9.22854	136.7117
25.39129854	92.64939582	11.28214	87.58032
23.97906986	87.23376529	9.458289	86.81358
20.46045669	97.25657193	8.112005	96.49497
22.46156751	82.38570647	8.888853	78.79156
21.77071352	93.46609834	8.242948	92.75515
21.88707224	81.11140848	8.613533	77.87295
17.69096273	95.82596706	6.326209	91.79071
24.01889317	93.97383561	7.834527	93.36754
21.81474715	93.22345951	8.754108	91.60497
24.01407406	102.6242774	9.291792	107.2151
22.79909767	107.6376647	8.388213	95.8595
17.68673077	93.38601955	5.780245	93.92247
15.48521661	93.1493858	5.532613	101.1401
16.51562494	107.9455162	5.534378	119.8331
23.19920693	103.0145527	8.723085	104.6996
22.63247907	96.29612141	8.383373	97.24776
23.64906649	97.07550284	8.4932	93.65256
16.00049226	92.90104351	5.271143	98.13755
14.56164935	94.27803007	3.94766	72.38145
Mean 22.61	97.24	9.847	96.71
SD 6.656	7.583	12.56	12.09

Dataset 15 - Raw data for Supplementary Figure 1 – Z-score analysis
