# Supplementary material for: Cardiac glycosides target barrier inflammation of the vasculature, meninges and choroid plexus
Source: Commun Biol. 2021 Feb 26;4:260. doi: 10.1038/s42003-021-01787-x (PMC7910294; doi:10.1038/s42003-021-01787-x)
Supplement: Supplementary file 18 — Reporting Summary [file 42003_2021_1787_MOESM18_ESM.pdf]

nature research

Corresponding author(s): Mike Dragunow

Last updated by author(s): Jan 6, 2021

Reporting Summary

Nature Research wishes to improve the reproducibility of the work that we publish. This form provides structure for consistency and transparency in reporting. For further information on Nature Research policies, see our Editorial Policies and the Editorial Policy Checklist.

Statistics

For all statistical analyses, confirm that the following items are present in the figure legend, table legend, main text, or Methods section.

n/a	Confirmed	
☐	☒	The exact sample size (n) for each experimental group/condition, given as a discrete number and unit of measurement
☒	☐	A statement on whether measurements were taken from distinct samples or whether the same sample was measured repeatedly
☐	☒	The statistical test(s) used AND whether they are one- or two-sided<br>*Only common tests should be described solely by name; describe more complex techniques in the Methods section.*
☒	☐	A description of all covariates tested
☐	☒	A description of any assumptions or corrections, such as tests of normality and adjustment for multiple comparisons
☐	☒	A full description of the statistical parameters including central tendency (e.g. means) or other basic estimates (e.g. regression coefficient) AND variation (e.g. standard deviation) or associated estimates of uncertainty (e.g. confidence intervals)
☒	☐	For null hypothesis testing, the test statistic (e.g. F, t, r) with confidence intervals, effect sizes, degrees of freedom and P value noted<br>*Give P values as exact values whenever suitable.*
☒	☐	For Bayesian analysis, information on the choice of priors and Markov chain Monte Carlo settings
☒	☐	For hierarchical and complex designs, identification of the appropriate level for tests and full reporting of outcomes
☒	☐	Estimates of effect sizes (e.g. Cohen's d, Pearson's r), indicating how they were calculated

Our web collection on statistics for biologists contains articles on many of the points above.

Software and code

Policy information about availability of computer code

Data collection	Cells were imaged using the ImageXpress® Micro XLS automated fluorescent microscope. Proteome profilers were imaged with LI-COR Odyssey® FC imaging system. Cytometric bead arrays were performed on an Accuri C6 Flow Cytometer (BD Biosciences). Human explant immunohistochemical sections were imaged with Metasystems V-slide scanning microscope, and confocal recording done using an FV1000 confocal microscope (Olympus).
Data analysis	Image analysis was performed using MetaXpress® software (Molecular Devices). Cytometric bead array analysis was performed using FCAP Array Software. Proteome profilers were quantified with Image Studio Software. Prism (Graphpad Software) was used for statistical testing and data are presented as mean +/- S.E.M. Multiple comparisons are noted in the corresponding figure legends. Heatmaps from proteome profilers were made in R Studio with gplots, heatmap.2.

For manuscripts utilizing custom algorithms or software that are central to the research but not yet described in published literature, software must be made available to editors and reviewers. We strongly encourage code deposition in a community repository (e.g. GitHub). See the Nature Research guidelines for submitting code & software for further information.

Data

Policy information about availability of data

All manuscripts must include a data availability statement. This statement should provide the following information, where applicable:
- Accession codes, unique identifiers, or web links for publicly available datasets
- A list of figures that have associated raw data
- A description of any restrictions on data availability

All data to support the findings in this study are presented in the figures and can be made available upon reasonable request to the corresponding author. There are restrictions on the identity of tissue sources only.

Field-specific reporting

Please select the one below that is the best fit for your research. If you are not sure, read the appropriate sections before making your selection.

☒ Life sciences ☐ Behavioural & social sciences ☐ Ecological, evolutionary & environmental sciences

For a reference copy of the document with all sections, see nature.com/documents/nr-reporting-summary-flat.pdf

Life sciences study design

All studies must disclose on these points even when the disclosure is negative.

Sample size	Sample sizes were based on previous in vitro experiments conducted in the lab, and published studies of drug screening. Some experiments were limited due to tissue availability.
Data exclusions	No data were excluded from the analysis.
Replication	Experiments were repeated in cells/tissue from at least 3 different individuals. All attempts at replication were successful.
Randomization	Randomization was not performed in this study as it was not applicable to the experiments involved. All cases tested were subject to the same in vitro conditions.
Blinding	Analysis of immunofluorescence was automated by high-throughput imaging and analysis software. Blinding was conducted during cytometric bead array experiments, and histological staining.

Reporting for specific materials, systems and methods

We require information from authors about some types of materials, experimental systems and methods used in many studies. Here, indicate whether each material, system or method listed is relevant to your study. If you are not sure if a list item applies to your research, read the appropriate section before selecting a response.

Materials & experimental systems

n/a	Involved in the study	
☐	☒	Antibodies
☒	☐	Eukaryotic cell lines
☒	☐	Palaeontology and archaeology
☒	☐	Animals and other organisms
☒	☐	Human research participants
☒	☐	Clinical data
☒	☐	Dual use research of concern

Methods

n/a	Involved in the study	
☒	☐	ChIP-seq
☐	☒	Flow cytometry
☒	☐	MRI-based neuroimaging

Antibodies

Antibodies used

Antibody/host/source/catalogue number/dilution>
alpha smooth muscle actin/mouse/Dako/IS611/1:100 IHC
CCL2(MCP-1)/rabbit/Abcam/ab9669/1:500 ICC
CD31/mouse/Dako/M0823/1:500 ICC, 1:100 IHC
CD68(KP1)/mouse/Abcam/ab955/1:500 IHC
CEBPdelta/mouse/Santa Cruz/sc-365546/1:500 ICC
Claudin-5/rabbit/Abcam/ab131259/1:5000 ICC
COL1A1/mouse/Santa Cruz/sc-293182/1:50 IHC
HLA-DR/mouse/Dako/M0775/1:1000 IHC
IBA1/goat/Abcam/ab5076/1:500 IHC
ICAM-1/mouse/Santa Cruz/sc-107/1:500 ICC
IL-6/goat/R&D Systems/AF-206/1/2000 ICC
NFkappaBp65/rabbit/Santa Cruz/sc-372/1:500 ICC
PDGFRbeta/goat/R&D Systems/AF-385/1:2000 ICC, 1:200 IHC
PDGFRbeta/rabbit/Cell Signaling/3169/1:100 IHC
PDLIM3/rabbit/ThermoFisher/Pa5-52099/1:50 IHC
PU.1/rabbit/Cell Signaling/2258/1:500 ICC
SMAD2/3/mouse/Santa Cruz/sc133098/1:500 ICC
STAT1/Rabbit/Cell Signaling/14994/1:500 ICC
Streptavidin Alexa Fluor 647//Invitrogen/S21374/2 mg/mL (1:500) IHC

Transthyretin, Pre-albumin/rabbit/Dako/A0002/1:1000 IHC
ZO-1/mouse/Invitrogen/339100/1:500 ICC
Lectin UEA-1//Sigma/L8262/1:1000 IHC
Hoechst 33258//Sigma/B2883/1:500 ICC, 1:10000 IHC

Validation

Antibodies used in this study have been either validated by the manufacturer, by siRNA knockdown or western blot.

Flow Cytometry

Plots

Confirm that:

☐ The axis labels state the marker and fluorochrome used (e.g. CD4-FITC).

☐ The axis scales are clearly visible. Include numbers along axes only for bottom left plot of group (a 'group' is an analysis of identical markers).

☐ All plots are contour plots with outliers or pseudocolor plots.

☐ A numerical value for number of cells or percentage (with statistics) is provided.

Methodology

Sample preparation

Describe the sample preparation, detailing the biological source of the cells and any tissue processing steps used.

Instrument

Identify the instrument used for data collection, specifying make and model number.

Software

Describe the software used to collect and analyze the flow cytometry data. For custom code that has been deposited into a community repository, provide accession details.

Cell population abundance

Describe the abundance of the relevant cell populations within post-sort fractions, providing details on the purity of the samples and how it was determined.

Gating strategy

Describe the gating strategy used for all relevant experiments, specifying the preliminary FSC/SSC gates of the starting cell population, indicating where boundaries between "positive" and "negative" staining cell populations are defined.

☐ Tick this box to confirm that a figure exemplifying the gating strategy is provided in the Supplementary Information.
